# Dysfunction of lipid storage droplet-2 suppresses endoreplication and induces JNK pathway-mediated apoptotic cell death in *Drosophila* salivary glands

**DOI:** 10.1038/s41598-022-08299-6

**Published:** 2022-03-11

**Authors:** Tran Duy Binh, Yen D. H. Nguyen, Tuan L. A. Pham, Kenichi Komori, Thanh Q. C. Nguyen, Masahide Taninaka, Kaeko Kamei

**Affiliations:** 1 Department of Functional Chemistry, Kyoto Institute of Technology, Kyoto, 606-8585 Japan; 2 Present Address: Faculty of Medical Technology, Hanoi Medical University, Hanoi, 100000 Vietnam; 3 Present Address: Department of Chemistry, Cantho University, Cantho, 900000 Vietnam

**Keywords:** Cell death, Cell division, Cell signalling, Developmental biology, Cell proliferation

## Abstract

The lipid storage droplet-2 (LSD-2) protein of *Drosophila* is a homolog of mammalian perilipin 2, which is essential for promoting lipid accumulation and lipid droplet formation. The function of LSD-2 as a regulator of lipolysis has also been demonstrated. However, other LSD-2 functions remain unclear. To investigate the role of LSD-2, we performed tissue-specific depletion in the salivary glands of *Drosophila* using a combination of the Gal4**-**upstream activating sequence system and RNA interference. LSD-2 depletion inhibited the entry of salivary gland cells into the endoreplication cycle and delayed this process by enhancing CycE expression, disrupting the development of this organ. The deficiency of LSD-2 expression enhanced reactive oxygen species production in the salivary gland and promoted JNK-dependent apoptosis by suppressing dMyc expression. This phenomenon did not result from lipolysis. Therefore, LSD-2 is vital for endoreplication cell cycle and cell death programs.

## Introduction

Lipid droplets—one of the major cellular organelles localized in the cytoplasm—are essential for energy homeostasis^1^. Most cells store lipids such as triacylglycerol (TAG) and cholesterol ester in lipid droplets, which are surrounded by a lipid monolayer membrane. Currently, there is a revival of interest in the study of lipid droplets, mostly due to the identification of their critical participation in metabolic diseases, and their role in the infectious cycle of pathogens targeting fat metabolic cells or lipid metabolic pathways for propagation^2^. Lipid droplets have also emerged as essential players in many intracellular trafficking pathways that are directly involved in lipid metabolism, as they can establish functional contact sites on the membranes of multiple organelles^3^. The role of lipid droplets in inflammatory responses has been recently elucidated^4^. In non-adipocytes, the main role of lipid droplets is protection from lipotoxicity by the storage of fatty acids in the form of neutral TAG. Fatty acids can be converted to lipid intermediates such as diacylglycerol, ceramides, and acyl-CoAs, which affect insulin signaling^5^. Thus, the dysfunction of lipid metabolism and lipid droplets has been implicated in the pathogenesis of numerous diseases, including common metabolic diseases, such as obesity and fatty liver disease, and cancer.

To regulate lipid droplet homeostasis, lipases, such as adipose triglyceride lipase (ATGL) and hormone-sensitive lipase (HSL), have to access their surface directly. Besides lipases, perilipins are lipid droplet-associated proteins that control intracellular lipid accumulation^6^. In mammals, the function of perilipins mostly concerns lipid metabolism as they regulate ATGL and HSL by either recruiting or preventing the access of these enzymes to lipid droplets^6,7^. Interestingly, these are evolutionarily conserved in a wide range of organisms, including mammals and the fruit fly *Drosophila melanogaster*^7^. In fatty tissues, lipolysis is indirectly regulated based on the phosphorylation of perilipins. In stimulated conditions, ATGL and the comparative gene identification 58 (CGI-58) complex are released by phosphorylated perilipins. This event leads to the full activation of ATGL and initiates the lipolysis process. Qi et al*.* identified the perilipin 1 locus as an obesity risk factor in humans^8^. Moreover, genetic variations in perilipins are associated with several metabolic disorders, such as type 2 diabetes and partial lipodystrophy^1,6^. In mice, perilipin 1 dysfunction leads to a leanness phenotype, exhibiting glucose intolerance and insulin resistance, whereas the complete lack of perilipin 1 on the background of a mutation in the leptin receptor gene inhibits obesity^9^. Perilipin 2 participates in the pathogenesis of diet-induced insulin resistance^10^. *Drosophila* encodes two perilipin family members—lipid storage droplets-1 and -2 [LSD-1/perilipin 1 (PLIN1) and LSD-2/perilipin 2 (PLIN2), respectively]^11^. LSD-1 is expressed on the surface of lipid droplets of different sizes, whereas LSD-2 mainly localizes to smaller lipid droplets^12^. In addition, the location of LSD-1 is exclusively on lipid droplets, whereas LSD-2 is present in the cytoplasm and lipid droplets^7^.

The cell cycle is a process in which cells can replicate and generate two new cells that maintain the same amount of genetic content as the mother cells. The most common cell cycle consists of four phases: G1, S, G2, and M, which involves mitosis. The endoreplication cycle or endocycle comprises only two phases, G- and S-phases^13^, thus generating cells with multiple copies of the genome called polyploid cells. This event generates either a cell that maintains separate nuclei and remains multinucleated, or a cell with an enlarged, single nucleus containing all DNA. Notably, the endocycle is evolutionarily conserved between fungi and humans. Polyploid cells are necessary to achieve normal organ size and functionality in many tissues and organs^14^. Polyploidy plays an essential role in organ development in many organisms as well as in cell differentiation. In addition, endocycles and polyploidy contribute to the protection of genome integrity, tissue homeostasis, and human diseases, especially cancer^15^. Therefore, understanding the endocycle is vital for determining the relationship between tissue homeostasis and diseases.

*Drosophila* has emerged as an excellent model for studying disciplines ranging from fundamental genetics to the development of tissues and organs. The salivary gland is an excellent experimental system for investigating endoreplication. In larval salivary gland cells, endoreplication is activated during the early stage of 3rd-instar larvae (from 82- to 84-h-old larvae after hatching of embryos [AEH]) and is completed during the late stage 3rd-instar larvae (from 110- to 112-h-old larvae AEH)^16^. The *Drosophila* salivary gland has two ventral ectodermal plates composed of approximately 100 cells each in the region of the presumptive posterior head, and consists of two major cell types: secretory and duct cells. Notch signaling plays an important role in switching cells from the mitotic cycle to the endocycle by activating fizzy-related/Cdh1 (Fzr/Cdh1) and suppressing cyclin-dependent kinase 1 (Cdk1) activity^17,18^. Moreover, cyclin E/cyclin-dependent kinase 2 (CycE)/Cdk2 oscillation may be achieved by the periodic expression of Dacapo, a p27^Cip/Kip^ homolog, and *Drosophila* p21-activated kinase, which inhibits CycE activity^19^. Transcription factor E2f1 is crucial as it is responsible for CycE transcription^20^. In *Drosophila*, dMyc plays a vital role in regulating cell size and larval growth and positively controls endoreplication^16^. The c-Jun N-terminal kinase (JNK) signaling pathway is a vital regulatory mechanism that controls stress-induced cell death, cell proliferation regulation, differentiation, metamorphosis, and tumor formation^19–21^. JNK belongs to the mitogen-activated protein kinase cascade and is initiated by various intrinsic and extrinsic signals, including tumor necrosis factor (TNF) family ligands^22,23^.

In the present study, to reveal the function of LSD-2 in the development of the *Drosophila* salivary gland, we analyzed the effect of *Lsd-2* knockdown (KD) on salivary glands from three differently aged 3rd-instar larvae: early stage (82- to 84-h-old AEH), middle stage (96-h-old AEH), and late stage (110- to 112-h-old AEH). We found that LSD-2 was essential for regulating cell death programs and endoreplication, thereby providing novel insights into its function in the JNK pathway.

## Results

### Localization of LSD-2 in the salivary glands of Drosophila

**Figure 1:**
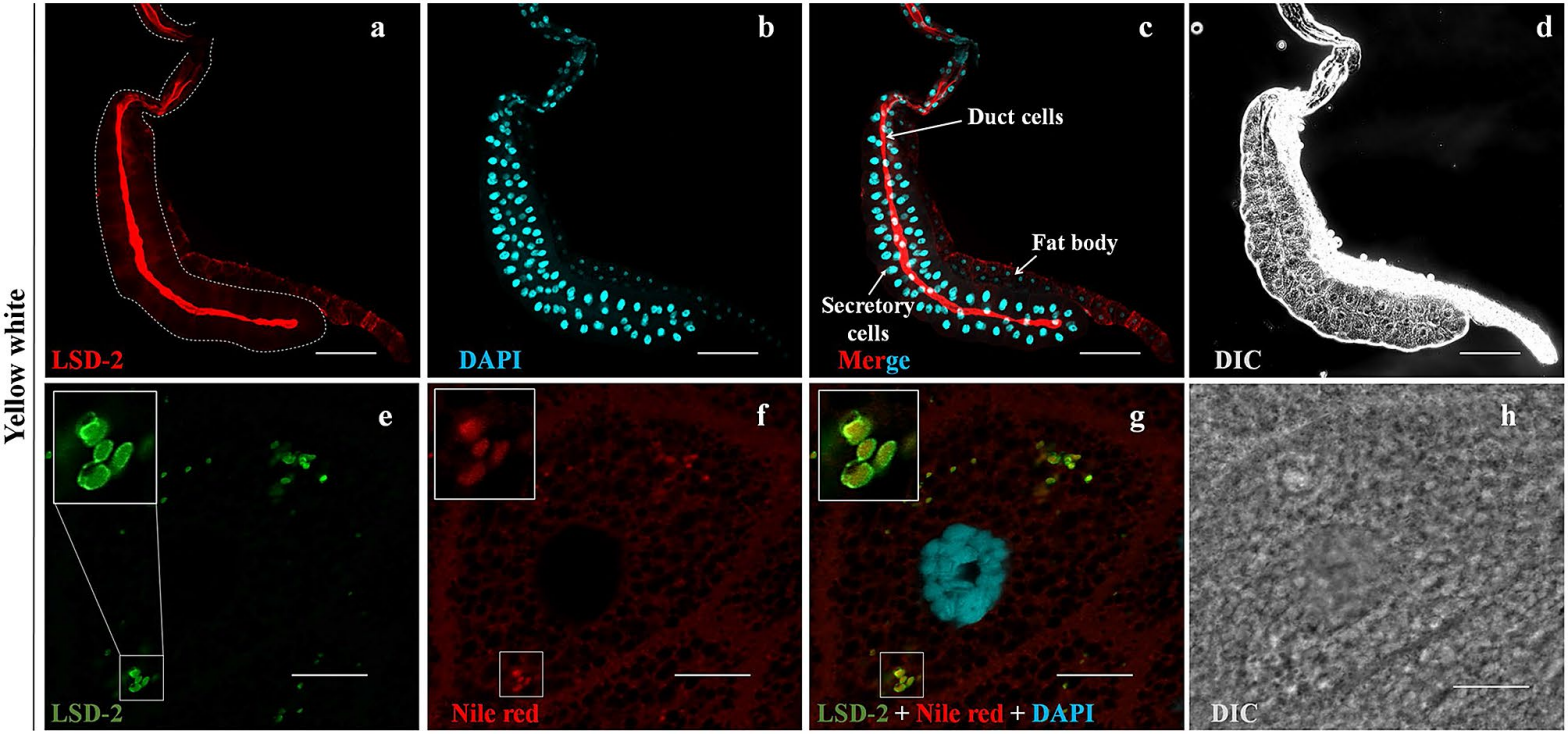
Localization of LSD-2 protein in the salivary gland. Salivary glands from middle stage 3rd-instar larvae of the yw were stained with anti-LSD-2 antibody (**a**,**e**) and DAPI (**b**,**f**). The LSD-2 and DAPI signals are merged in (**c**,**g**). DIC images of whole salivary glands from 3rd-instar larvae (**d**) and secretory cells (**h**) are shown. LSD-2 was highly expressed in the lumen of the tube in which duct cells reside (**a**) and in secretory cells (**e**). Lipid droplets were stained with Nile red (**f**). Merged images of LSD-2, Nile red, and DAPI signals indicate LSD-2 localization on the intracellular lipid droplets (**g**). In (**e**–**g**), each panel on the left side includes the image at higher magnifications of the boxed region under each panel. The images in the figure are representative images of 30 salivary glands. The salivary gland is demarcated with a dotted line (**a**). Scale bar: 200 µm (**a**–**d**) and 5 µm (**e**–**h**). *DIC* differential interference contrast.

LSD-2 is expressed during all stages of the *Drosophila* lifespan, especially in the fat body tissue of 3rd-instar larvae^24^. To determine the location of LSD-2 in the salivary glands, we stained the salivary glands of the middle stage 3rd-instar larvae (96-h-old AEH) of the yellow-white (yw) strain with an anti-LSD-2 antibody. The results showed that LSD-2 signals were highly detected in the lumen of the tube in which the duct cells resided (Fig. 1a–c), and in the intracellular lipid droplets of secretory cells marked with Nile Red (Fig. 1e–g). Furthermore, we overexpressed GFP using *C147*-GAL4 drivers to confirm the stage of salivary glands in *Drosophila* larvae when GAL4 was expressed. We found that stronger GFP signals were detected at the 3rd-instar larvae stage driven by *C147*-GAL4, indicating that *C147*-GAL4 is active in the 3rd-instar larvae (Supplementary Figs. S1–S2).

### Dysfunction of LSD-2 disrupted salivary gland development

**Figure 2:**
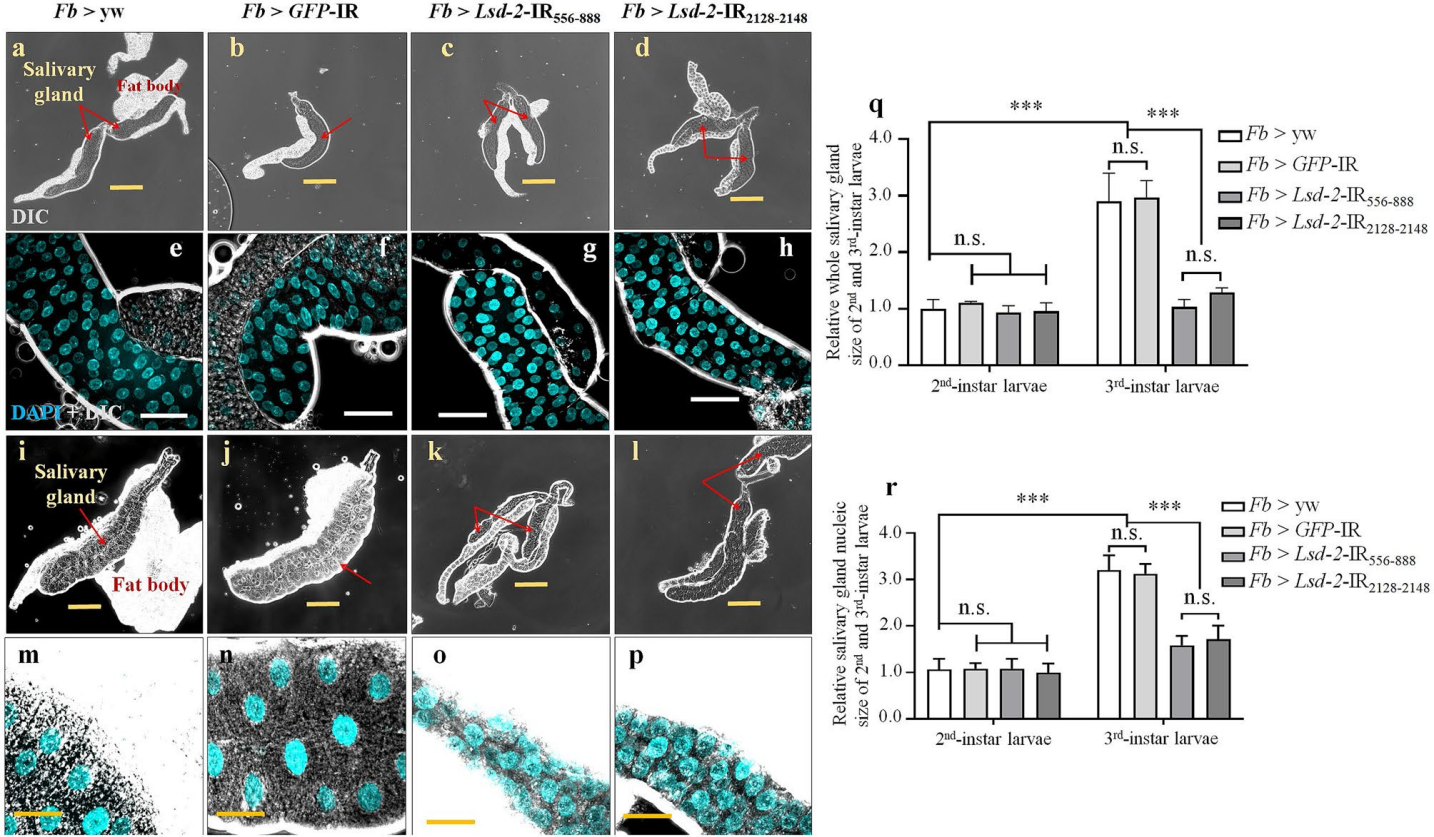
Knockdown of *Lsd-2* induced a smaller salivary gland in 3rd-instar larvae. DIC images of salivary glands from 2nd-instar larvae (**a**–**d**) and 3rd-instar larvae (**i**–**l**) are shown. Control flies were obtained by mating *Fb*-GAL4 driver flies with the yw strain (**a**,**e**,**i**,**m**) and with *GFP*-IR (**b**,**f**,**j**,**n**). The salivary gland size of *Lsd-2* KD flies [*Fb* > *Lsd-2*-IR_556-888_ (**c**,**g**,**k**,**o**) and *Fb* > *Lsd-2*-IR_2128–2148_ (**d**,**h**,**l**,**p**)] were normal in 2nd-instar larvae (**c**–**d**), but smaller in 3rd-instar larvae (**k**,**l**) than those of the control flies (**a**,**b**,**i**,**j**). The relative sizes of salivary glands of 2nd-instar and 3rd-instar larvae of *Lsd-2* KD flies compared to those of control flies are shown (**q**). The salivary gland cells of 2nd-instar and 3rd-instar larvae from controls and *Lsd-2* KD flies were stained with DAPI to visualize the DNA (**e**–**h**,**m**–**p**). The nucleic size of salivary gland secretory cells from *Lsd-2* KD flies was normal in 2nd-instar larvae (**g**,**h**), but smaller in 3rd-instar larvae (**o**,**p**) compared to those of control flies (**e**,**f**,**m**,**n**). The relative nucleic size of salivary gland secretory cells from 2nd-instar and 3rd-instar larvae of *Lsd-2* KD flies compared to those of control flies are shown (**r**). The red arrows indicate salivary glands. The perimeter of salivary gland size and the nucleus cell size of the salivary gland were analyzed using ImageJ software (*n* = 20 for each genotype). The statistical significance of the differences in parameters between *Lsd-2* KD and control flies was evaluated using one-way analysis of variance (ANOVA). Scale bar, 200 µm (**a**–**d**,**i**–**l**), and 50 µm (**e**–**h**,**m**,**n**). n.s., no significant; ***p < 0.001. Genotypes: (**a**,**e**,**i**,**m**)+; *Fb*-GAL4/+; +, (**b**,**f**,**j**,**n**)+; *Fb*-GAL4/+; UAS-*GFP*-IR/+, (**c**,**g**,**k**,**o**)+; *Fb*-GAL4/UAS*-Lsd-2*-IR_556–888_/;+, (**d**,**h**,**l**,**p**)+; *Fb*-GAL4/+; UAS*-Lsd-2-*IR_2128–2148_/+. *DIC* differential interference contrast.

To determine LSD-2 function in the development of salivary glands, two RNAi fly lines, UAS-*Lsd-2*-IR_556-888_ and UAS-*Lsd-2*-IR_2128-2148_, were crossed with *Fb*-GAL4, which expresses GAL4 in embryonic, larval salivary glands, and fat bodies^25^. We excluded the effects of off-targets using these two different KD flies. We measured the perimeter of each lobe of the salivary gland to assess its size. As shown in Fig. 2, the control flies displayed an enlargement in the salivary glands approximately 2.9-fold from the 2nd-instar larval stage to the 3rd-instar larval stage, whereas the KD flies did not. Therefore, both KD strains possessed significantly smaller salivary glands in the 3rd-instar larvae (44% and 36% smaller in the sizes of *Fb* > *Lsd-2* IR_556-888_ and *Fb* > *Lsd-2* IR_2128–2148,_ respectively) than those in control flies (*Fb* > yw and *Fb* > *GFP*-IR). The perimeter of the nucleic area of the salivary gland secretory cells was also calculated to assess the nuclei of salivary gland cells. The control flies showed a 3.0-fold higher average from the 2nd-instar to the 3rd-instar larval stage; however, the two KD flies (*Fb* > *Lsd-2* IR_556–888_ and *Fb* > *Lsd-2* IR_2128–2148_) showed increased only 1.7-fold and 1.5-fold, respectively, on average.

**Figure 3:**
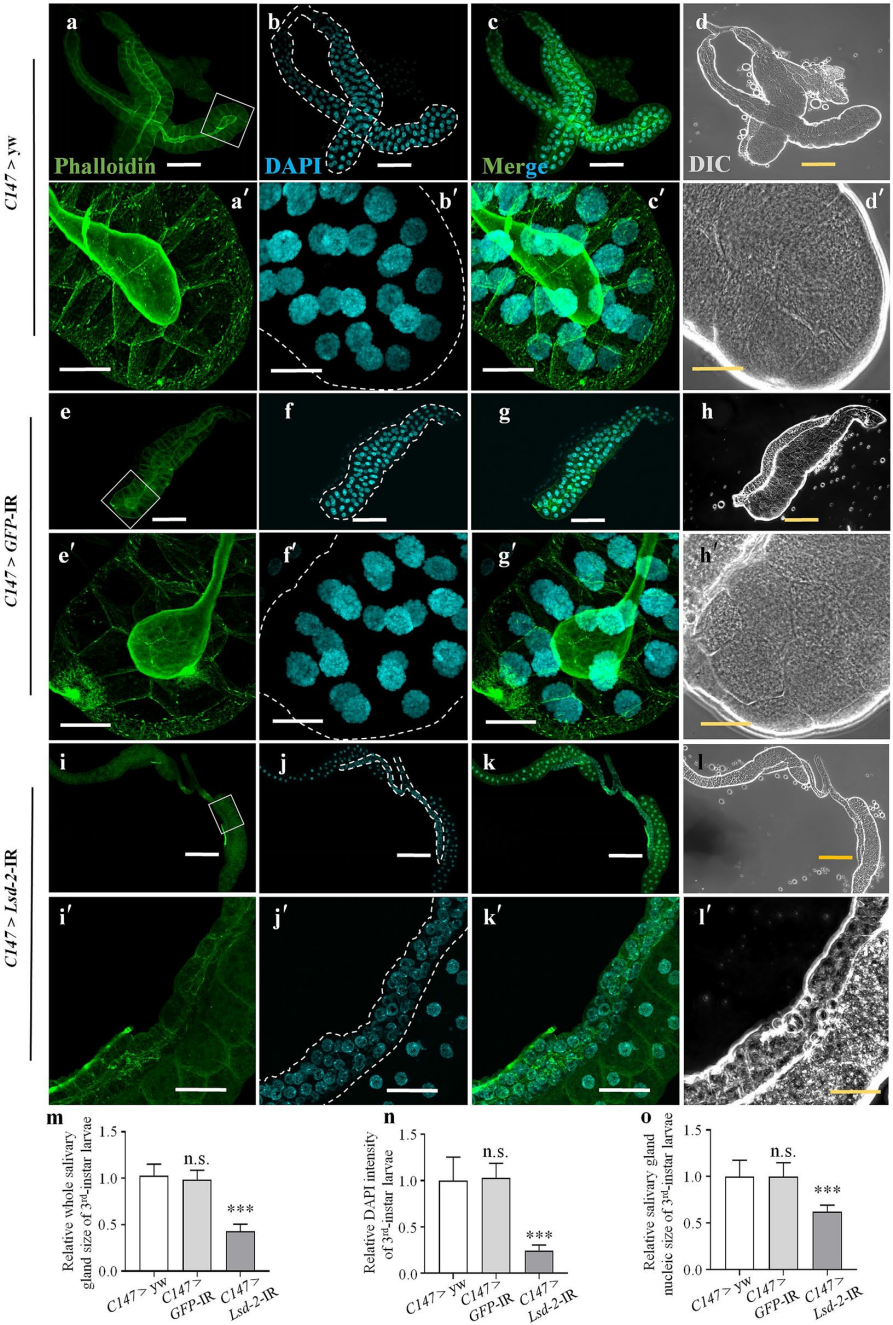
Knockdown of *Lsd-2* suppressed the endoreplication of salivary gland cells in middle stage 3rd-instar larvae. Control (*C147* > yw and *C147* > *GFP*-IR) flies were obtained by crossing the *C147*-GAL4 driver with the yw strain and RNAi GFP strain, respectively, whereas *Lsd-2* KD (*C147* > *Lsd-*2-IR_556–888_) flies were established using the *C147*-GAL4 driver. The salivary glands of 3rd-instar larvae from the controls and *Lsd-2* KD flies were stained with phalloidin to label the cell skeleton (**a**,**a**′,**e**,**e**′,**i**,**i**′), and DAPI to visualize the DNA (**b**,**b**′,**f**,**f**′,**j**,**j**′). Merged images of phalloidin and DAPI signals (**c**,**c**′,**g**,**g**′,**k**,**k**′) are shown. DIC images of whole salivary glands from 3rd-instar larvae (**d**,**d**′,**h**,**h**′,**l**,**l**′) are shown. *Lsd-2* KD flies (**i**) possessed small salivary glands compared to those of control flies (**a**,**e**). The panels (**a**′,**e**′,**i**′) are higher magnifications of the boxed regions in (**a**,**e**,**i**), respectively. The relative size of the salivary gland (*n* = 16 for each genotype) (**m**), relative DAPI intensity (*n* = 30 for each genotype) (**n**), and relative nucleic size of salivary gland cells (*n* = 30 for each genotype) (**o**) of 3rd-instar larval salivary glands of *Lsd-2* KD flies, compared to those of the control flies, are shown. The sizes of the salivary glands and nuclei were analyzed using the ImageJ software. DAPI intensity was analyzed using MetaMorph software. The salivary glands are demarcated with dotted lines (**b**,**b**′,**f**,**f**′,**j**,**j**′). The statistical significance of the differences in parameters between *Lsd-2* KD and control flies was evaluated using one-way analysis of variance (ANOVA). Scale bar, 200 µm (**a**–**l**); 50 µm (**a′**–**l′**); n.s., no significant; ***p < 0.001. Genotypes: (**a**–**d**,**a′**–**d′**)+; *C147*-GAL4/+; +, (**e**–**h** and **e′**–**h′**)+; *C147*-GAL4/+; UAS-*GFP*-IR/+, (**i**–**l**,**i′**–**l′**)+; *C147*-GAL4/UAS*-Lsd-2-*IR_556-888_/; +. *DIC* differential interference contrast.

The other KD flies were established by crossing the UAS-*Lsd-2*-IR_556–888_ strain with the *C147*-GAL4 driver. The obtained flies possessed smaller and underdeveloped salivary glands during the middle 3rd-instar larval stage (Fig. 3i–l,m) compared to the control flies (Fig. 3a–h,m). Although the salivary glands of the *Lsd-2* KD flies contained approximately the same number of cells as the controls (100–110 cells/lobe), the salivary gland cells from the 3rd-instar larvae of the *Lsd-2* KD flies were represented by a disrupted cell skeleton, smaller nuclei, and reduced DNA content (Fig. 3i′–l′,n,o) compared to those of the control flies (Fig. 3a′–h′,n,o). Therefore, LSD-2 depletion suppressed endoreplication, resulting in decreased polyteny levels.

A flip-out experiment was performed to confirm the targeted effects of *Lsd-2* dsRNA (*Lsd-2*-IR_556–888_) on LSD-2 expression in the salivary gland. Cells expressing *Lsd-2* dsRNA were marked with green fluorescent protein-GFP (green). Immunostaining showed a decreased level of LSD-2 signal (red) in the GFP-marked cells, confirming the specific KD of *Lsd-2* in the salivary glands (Supplementary Fig. S3). Furthermore, quantitative reverse transcription PCR (RT-qPCR) and immunostaining showed that the mRNA and protein levels of *Lsd-2* were significantly decreased in the salivary glands of the KD flies, *C147* > *Lsd-2*-IR (Supplementary Fig. S4). These results confirmed the specific KD of *Lsd-2* in the salivary glands after *Lsd-2* dsRNA expression.

### LSD-2 KD phenotype is not related to lipolysis

**Figure 4:**
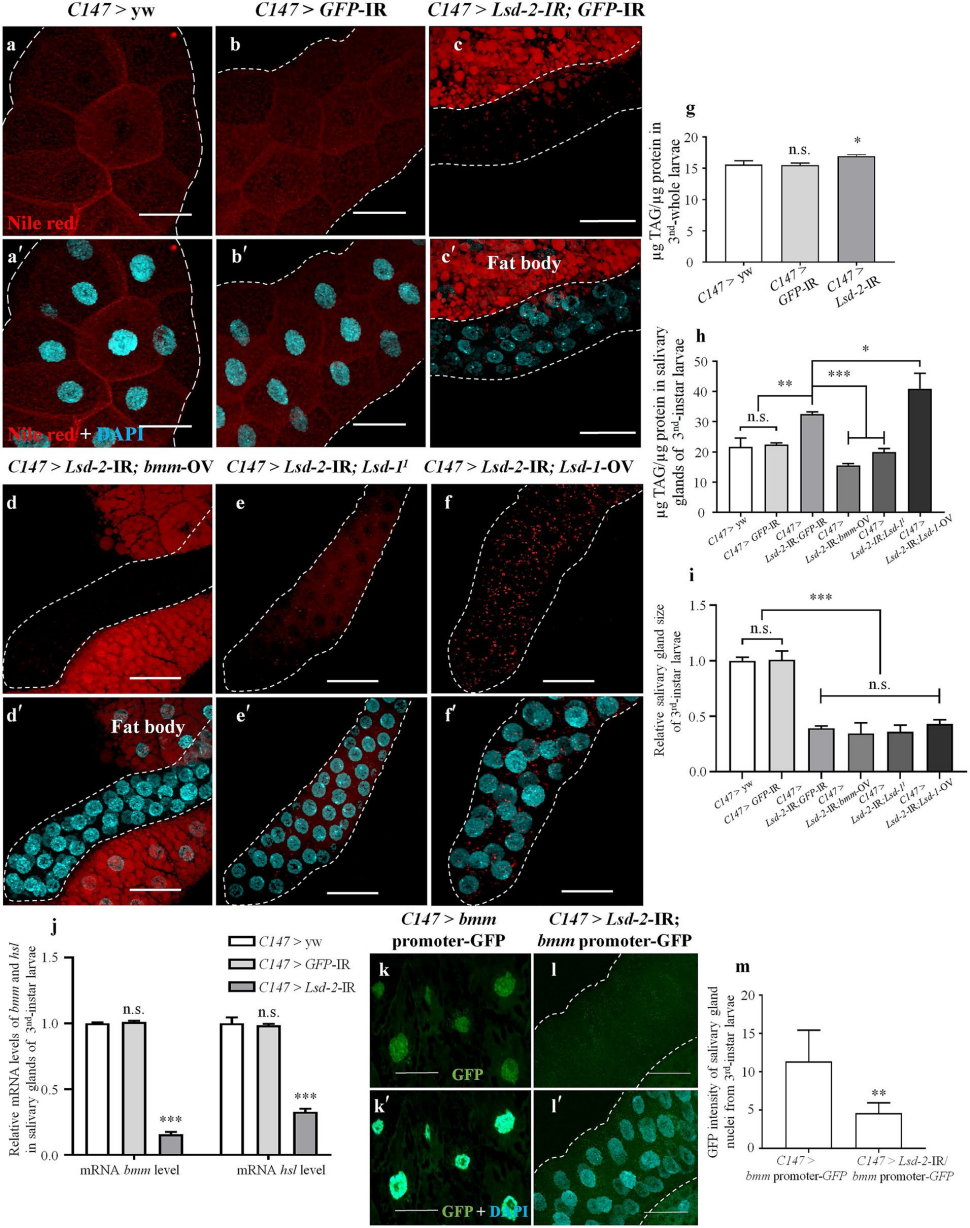
Knockdown of *Lsd-2* caused misregulated lipolysis in the 3rd-instar larval salivary gland. Salivary glands from the middle stage 3rd-instar larvae of controls and *Lsd-2* KD flies were stained with Nile Red (**a**–**c**) to visualize lipid droplets. The elevation of intracellular lipid droplets stained with Nile Red in *Lsd-2* KD was suppressed by overexpression of BMM (*C147* > *Lsd-2*-IR; *bmm*-OV, (**d**) or lack of LSD-1 expression (*C147* > *Lsd-2*-IR; *Lsd-1*^*1*^*,* (**e**). In contrast, the elevation of intracellular lipid droplets was enhanced by overexpression of LSD-1 (*C147* > *Lsd-2*-IR; *Lsd-1*-OV, (**f**) and remained unchanged by the expression of GFP dsRNAi (**c**). Merged images of DAPI and Nile Red staining are shown (**a′**–**f′**). Relative TAG levels in the whole body of controls and *Lsd-2* KD (**g**), and salivary glands of *Lsd-2* KD, *Lsd-2*-IR; *bmm*-OV, *Lsd-2*-IR; *Lsd-1*^*1*^ and *Lsd-2*-IR; *Lsd-1*-OV 3rd-instar larvae, to those in the controls (**h**), were analyzed (*n* = 4 for each genotype). The relative salivary gland sizes of *Lsd-2* KD, *Lsd-2*-IR; *bmm*-OV, *Lsd-2*-IR; *Lsd-1*^*1*^ and *Lsd-2*-IR; *Lsd-1*-OV 3rd-instar larvae, to those in the controls were analyzed using ImageJ software (**i**, *n* = 30 for each phenotypes). The relative *bmm* and *hsl* mRNA levels in the salivary glands of 3rd-instar larvae of control and *Lsd-2* KD flies were analyzed using RT-qPCR (**j**, *n* = 4). Fluorescence microscopy images of 3rd-instar larvae salivary glands of GFP-expression flies under regulation of the *bmm* promoter, controls (*C147* > *bmm* promoter-GFP, **k**), and KD flies (*C147* > *Lsd-2*-IR; *bmm* promoter-GFP, **l**) are shown. The merged images of GFP and DAPI are shown (**k′** and **l′**). The GFP intensity in the nuclei of 3rd-instar larval salivary glands of control and *Lsd-2* KD flies were analyzed using MetaMorph software (**m**, *n* = 12 for each genotype). The salivary gland is demarcated with a dotted line. The statistical significance of the difference was evaluated using one-way analysis of variance (ANOVA) and Student’s *t*-test. Scale bar, 50 µm; n.s., no significant; *p < 0.05; **p < 0.01; ***p < 0.001. Genotypes: (**a**,**a′**)+; *C147*-GAL4/+; +, (**b**,**b′**)+; *C147*-GAL4/+; UAS-*GFP*-IR/+, (**c**,**c′**) + ; *C147*-GAL4/UAS*-Lsd-2-*IR_556–888_; UAS-*GFP*-IR/+, (**d**,**d′**)+; *C147*-GAL4/ UAS*-Lsd-2-*IR_556–888_; UAS-*bmm*-OV/+, (**e**,**e′**)+; *C147*-GAL4/UAS*-Lsd-2-*IR_556–888_; *Lsd-1*^*1*^/+, (**f**,**f′**)+; *C147*-GAL4/UAS*-Lsd-2-*IR_556–888_; UAS-*Lsd-1*-OV/+. (**k**,**k′**)+; *C147*-GAL4/+; *bmm* promoter-GFP/+, (**l**,**l′**)+; *C147*-GAL4/ UAS*-Lsd-2-*IR_556–888_; *bmm* promoter-GFP/+.

To confirm whether *Lsd-2* KD phenotypes were caused by a deficiency of energy stocks, we measured the TAG content of whole bodies and salivary glands of middle stage 3rd-instar larvae of controls (*C147* > yw and *C147* > *GFP*-IR) and *Lsd-2* KD (*C147* > *Lsd-2*-IR) flies. *Lsd-2* KD had a significant increase in TAG levels in the salivary glands and whole body (Fig. 4g,h). The number of lipid droplets stained with Nile Red was also elevated in the salivary glands of KD flies (Fig. 4c) compared to that in the salivary glands of control flies (Fig. 4a,b). We hypothesized that the extreme accumulation of intracellular lipid droplets might indirectly affect the development of salivary glands due to excess lipotoxicity. To clarify this point, we investigated the involvement of lipases, Bummer (BMM), which is homologous to ATGL in humans and HSL, and LSD-1 in the increase of intracellular lipid droplets in *Lsd-2* KD salivary glands. First, we analyzed *bmm* and *hsl* mRNA expression during the middle stage of 3rd-instar larvae of the control and *Lsd-2* KD flies using RT-qPCR. The results demonstrated that the levels of both lipase mRNAs in KD flies were significantly lower than those in the control flies (Fig. 4j), suggesting that the excessive accumulation of intracellular lipid droplets may result from the suppression of lipolysis in the salivary gland by deficient LSD-2 expression. Next, we evaluated BMM expression using a reporter assay by crossing *Lsd-2* KD flies with *bmm* promoter-*GFP* flies^26^, which was established by introducing the *GFP* gene as a downstream reporter of the *bmm* promoter, and GFP intensity was measured using MetaMorph software. The results showed that the KD of *Lsd-2* in the salivary gland driven by *C147*-GAL4 resulted in a significant reduction in BMM expression (Fig. 4k,l,m). Moreover, *Lsd-2* KD was established, in which *bmm* was overexpressed, to observe the *Lsd-2* KD phenotypes. The excessive accumulation of intracellular lipid droplets in KD flies was decreased by *bmm* overexpression (Fig. 4d,h). These data reinforced our hypothesis that lipolysis was interrupted in the salivary glands of *Lsd-2* KD. Both lipases are regulated by LSD-1 activation^27^. Therefore, we investigated the association between LSD-1 and *Lsd-2*-KD phenotypes by observing the loss-of-function or overexpression of LSD-1 in *Lsd-2* KD flies. The homozygous *Lsd-1* mutant, *Lsd-1*^*1*^, has a deletion from position − 509 to + 82 containing the putative start site of LSD-1, causing the loss of LSD-1 protein expression, and the LSD-1 overexpression strain was generated by cloning a fragment of the *Lsd-1* complement DNA covering the LSD-1 open reading frame, enhancing LSD-1 protein expression^7^. We established *Lsd-2* KD flies on the background of the *Lsd-1*^*1*^ mutant or in which *Lsd-1* was overexpressed by the *C147*-GAL4 driver to check whether the *Lsd-2* phenotypes could be rescued. Consistent with the *bmm* overexpression results, the accumulation of intracellular lipid droplets was suppressed by the deficient expression of *Lsd-1* (Fig. 4e,h), whereas the overexpression of LSD-1 significantly enhanced the accumulation of lipid droplets (Fig. 4f,h). Abnormal salivary gland development, such as smaller salivary glands and their nuclei induced by *Lsd-2* KD, was not rescued by increasing or decreasing the expression of BMM and LSD-1 (Fig. 4i). These data imply that disrupted salivary gland development in *Lsd-2* KD flies may not be caused by deficient TAG storage or lipotoxicity.

### Dysfunction of LSD-2 delayed endoreplication cycle progression

**Figure 5:**
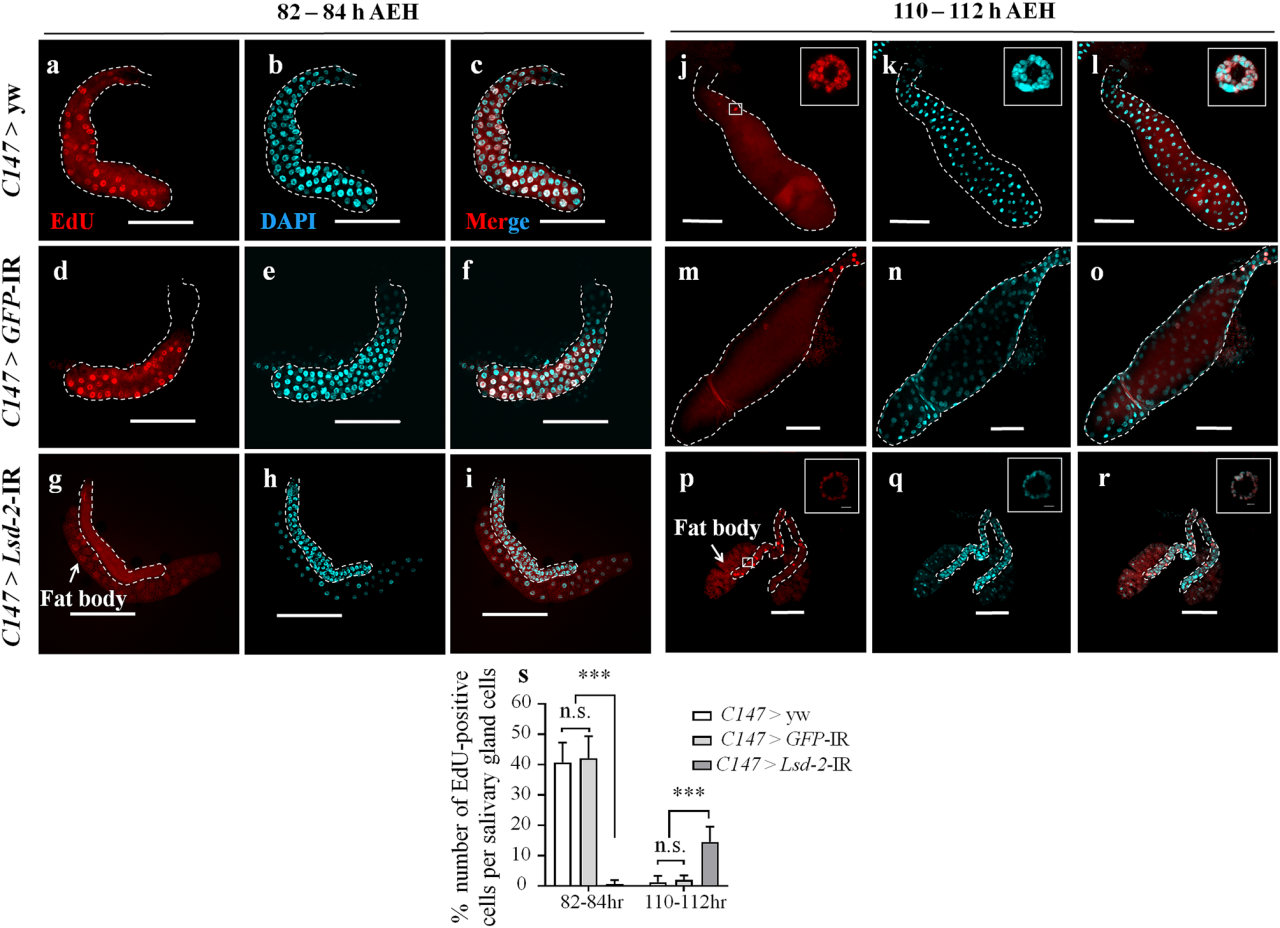
Reduction of LSD-2 led to a delayed S-phase in the salivary gland. Salivary glands from two age groups of 3rd-instar larvae, 82- to 84-h AEH (**a**–**i**) and 110- to 112-h AEH (**j**–**s**), of controls (*C147* > yw and *C147* > *GFP*-IR), and *Lsd-2* KD (*C147* > *Lsd-*2-IR_556–888_) flies were stained with click-iT™ EdU Alexa Fluor™ 594 (**a**,**d**,**g**,**j**,**m**,**q**) and DAPI (**b**,**e**,**h**,**k**,**n**,**q**). Merged images of EdU and DAPI staining are shown (**c**,**f**,**i**,**l**,**o**,**s**). Each panel (**j**–**l**,**p**–**r**) includes higher magnifications of the boxed regions shown in the left panels (**j**–**l**,**p**–**r**). The dotted line indicates the salivary gland area. The number of EdU-positive cells in 3rd-instar larvae was analyzed using MetaMorph software (**s**, *n* = 12 for each genotype). The statistical significance of the differences between *Lsd-2* KD and control flies was evaluated using one-way analysis of variance (ANOVA). Scale bar, 200 µm or 5 µm (large magnification images); n.s., not significant; ***p < 0.001. Genotypes: (**a**–**c**,**j**–**l**)+; *C147*-GAL4/+; +, (**d**–**f**,**m**–**o**)+; *C147*-GAL4/+; UAS-*GFP*-IR/+, (**g–i**,**p–r**)+; *C147*-GAL4/UAS*-Lsd-2-*IR_556-888_; +.

The reduction in polyteny levels in the salivary glands of *Lsd-2* KD could be caused by either fewer endoreplication cycles or an abrupt blockage of replication at the earlier stages of endoreplication. To prove this, we analyzed 5-ethynyl-20-deoxyuridine (EdU) incorporation into secretory cells^28^ at two different stages of 3rd-instar larvae: the early stage and the late stage^16^. The control flies showed 40.7% EdU-positive cells/salivary gland cells on average at the early stage, but no EdU-positive cells among most of the cells at the late stage, except for some cells in the proximal region (Fig. 5a,d,j,m). For *Lsd-2* KD flies, 0.7% of cells in the salivary gland were EdU-positive at the early stage, and EdU incorporation was continued (14.5% EdU-positive cells) even at the late stage (Fig. 5g,p), suggesting that LSD-2 depletion prevented the salivary gland cells from entering the S phase and caused incomplete termination of endoreplication at the expected time points. These results imply that the *Lsd-2* KD cells in the salivary glands displayed a late endoreplication process.

Oscillation of the CyclinE (CycE)-CDK2 complex is essential for endoreplication cycles and the transition from G1 to S phase^29^. We speculated that the G1/S checkpoint in the *Lsd-2* KD salivary gland might be activated by the dysregulated expression of CycE. To test this hypothesis, we analyzed the mRNA levels of *cyclin E* in the salivary glands of early 3rd-instar larvae of *Lsd-2* KD and control (*C147* > yw) strains by RT-qPCR. As hypothesized, CycE mRNA levels were significantly increased in KD flies (Supplementary Fig. S5g). Next, we established an overexpression strain of *CycE*, and the protein levels of CycE in early 3rd-instar larval salivary glands were examined by staining with the anti-CycE antibody. The results demonstrated that the CycE protein was detected more strongly than the control flies (Supplementary Fig. S5a–c). Thus, we confirmed the reactivity of the CycE antibodies. Immunostaining showed higher expression levels of CycE protein in the *Lsd-2* KD flies (Supplementary Fig. S5d).

Furthermore, we established a double KD strain of *Lsd-2* and *CycE* by a combination of *C147*-GAL4 and RNAi to determine whether the phenotype of the *Lsd-2* KD flies could be rescued. The phenotype of the smaller salivary glands in *Lsd-2* KD flies was partially rescued by *CycE* KD (Supplementary Fig. S5e–e′′,f–f′′,h). Taken together, reduced LSD-2 expression disrupts the regulation of CycE expression, leading to delayed S-phase progression in the endocycle.

### LSD-2 dysfunction causes reactive oxygen species (ROS) and induces activation of the JNK pathway

**Figure 6:**
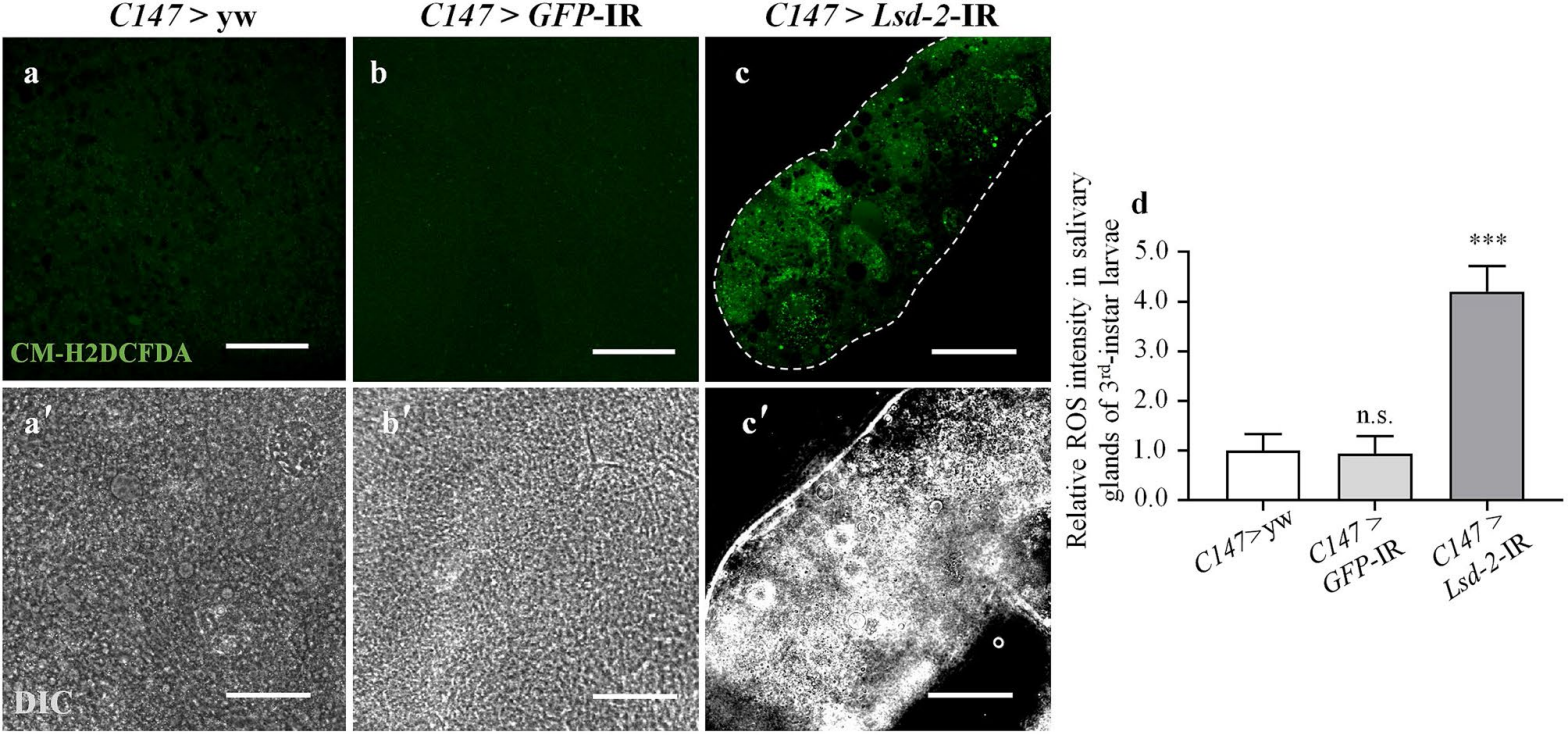
Knockdown of *Lsd-2* induces overproduction of ROS in salivary gland. ROS generation in the salivary glands of middle stage 3rd-instar larvae of controls (**a**,**b**) and *Lsd-2* KD (**c**) was detected using CM-H_2_DCFDA. The controls (*C147* > yw and *C147* > *GFP*-IR) showed unclear ROS signals in salivary gland cells, while the *Lsd-2* KD (*C147* > *Lsd-2*-IR) showed increased ROS signals. DIC images of salivary glands from 3rd-instar larvae (**a′**–**c′**) are shown. Relative ROS intensity in the salivary glands was analyzed using MetaMorph software (**d**, *n* = 16 for each phenotype). The images in the figure are representative images of 16 salivary glands. The dotted line indicates salivary gland area. The statistical significance of differences in ROS intensity between control and *Lsd-2* KD flies was evaluated using one-way analysis of variance (ANOVA). n.s., no significant; ***p < 0.001. Scale bar, 50 µm. Genotypes: (**a**,**a′**)+; *C147*-GAL4/+; +, (**b**,**b′**) +; *C147*-GAL4/UAS*-GFP-*IR/+; +, (**c**,**c′**)+; *C147*-GAL4/UAS*-Lsd-2-*IR_556–888_/+; + . *DIC* differential interference contrast.

Previous study has indicated that depletion of LSD-2 increases reactive oxygen species (ROS) production^30^. To detect intracellular ROS production in *Lsd-2* KD flies, we used the non-fluorescent substrate CM-H_2_DCFDA, which can be oxidized by ROS to an intracellular green fluorescent product. Figure 6c,d showed that much stronger fluorescent signals were detected in the salivary gland cells of *Lsd-2* KD larvae, implying that deficient LSD-2 led to increased ROS production in the salivary gland cells.

**Figure 7:**
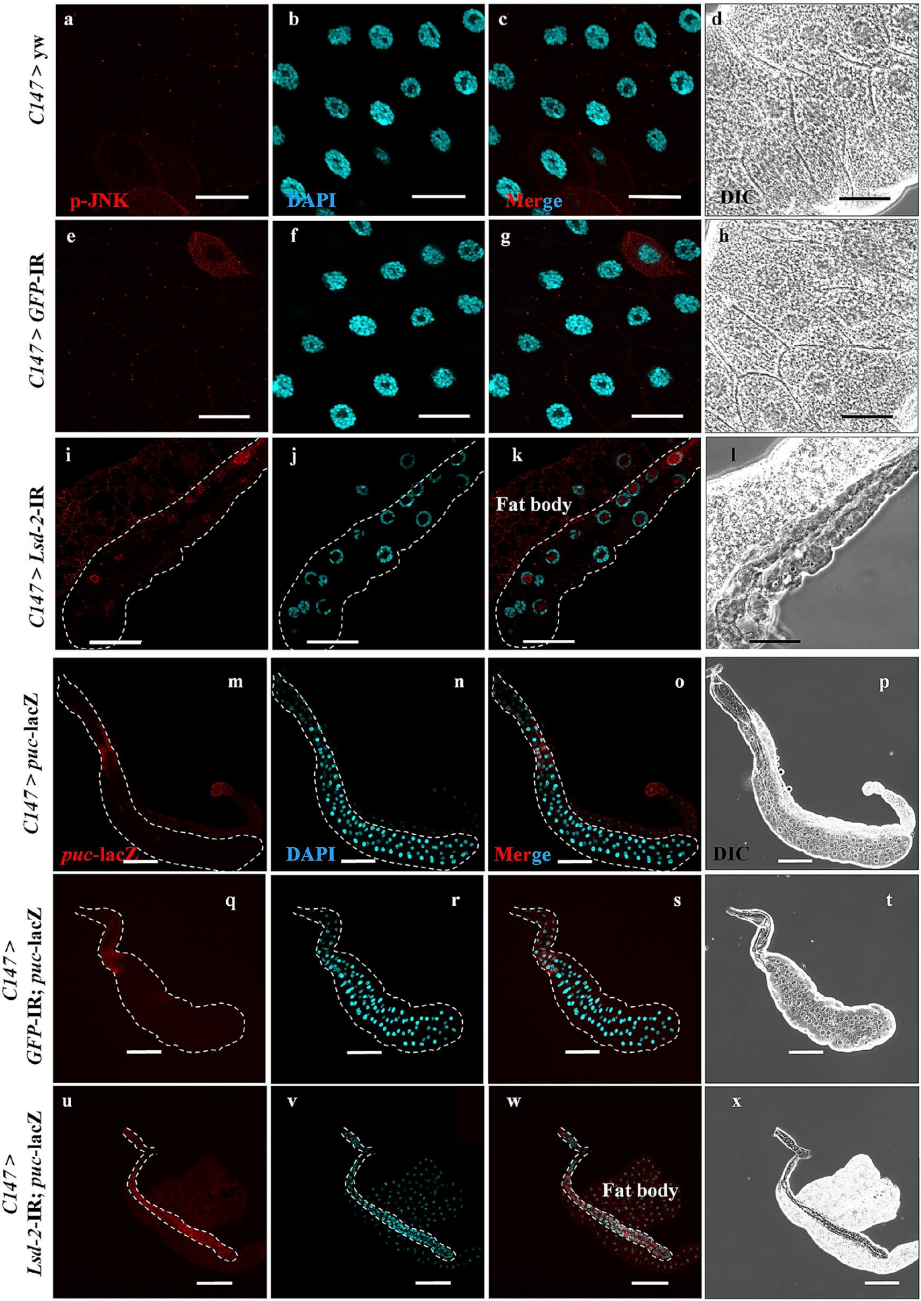
*Lsd-2* KD induced the activation of JNK pathway in the salivary gland. Salivary glands from middle stage 3rd-instar larvae of controls and *Lsd-2* KD flies were stained with rabbit anti-p-JNK antibody (**a**,**e**,**i**). The controls and *Lsd-2* KD flies, which were the introduced *puc-*lacZ gene, were stained with chicken anti-lacZ (**m**,**q**,**u**). Both were followed by reaction with anti-rabbit or chicken IgG Alexa Fluor™ 594 antibody and DAPI (**b**,**f**,**j**,**n**,**r**,**v**). Merged images of DAPI and rabbit anti-p-JNK (**c**,**g**,**k**) or anti-lacZ (**o**,**s**,**w**) antibody staining are shown. DIC images of salivary glands from 3rd-instar larvae (**d**,**h**,**l**,**p**,**t**,**x**) are shown. The dotted line indicates the salivary gland area. The images in the figure are representative images of 26 salivary glands. Scale bar, 50 μm (**a–l**) or 200 μm (**m–x**). Genotypes: (**a–d**)+; *C147*-GAL4/+; +, (**e–h**) +; *C147*-GAL4/+; UAS*-GFP-*IR/+, (**i–l**) +; *C147*-GAL4/UAS*-Lsd-2-*IR_556–888_; +, (**m–p**) +; *C147*-GAL4/+; UAS-*puc*-lacZ/+, (**q–t**) +; *C147*-GAL4/+; UAS*-GFP-*IR/UAS-*puc*-lacZ, (**u–x**) +; *C147*-GAL4/UAS*-Lsd-2-*IR_556–888_; UAS-*puc*-lacZ/+. *DIC* differential interference contrast.

During the pupal stages, JNK is activated and promotes disintegration of salivary glands^31,32^. Therefore, we hypothesized that LSD-2 dysfunction might activate the JNK pathway earlier. Figure 7a–l showed that LSD-2 depletion induced the translocation of p-JNK from the cytoplasm into the nucleus of salivary gland cells of middle stage 3rd-instar larvae. Fat body cells, used as an internal control, showed no change in the translocation of p-JNK between the control (Fig. 7a–h) and *Lsd-2* KD flies (Fig. 7i–l). Next, we examined JNK activation indirectly by detecting the expression of puckered (*puc*) using a lacZ reporter, which was previously reported to be highly expressed in *Drosophila* JNK-activated cells (Fig. 7m–x). Consistent with the above results, the signal of *puc*-lacZ was significantly elevated in the salivary glands of *Lsd-2* KD flies (Fig. 7u–x) compared to controls (Fig. 7m–t). These results suggest that LSD-2 expression is essential for regulating the activation of the JNK signaling pathway.

### KD of Lsd-2 led to DNA damage-induced apoptotic cell death

**Figure 8:**
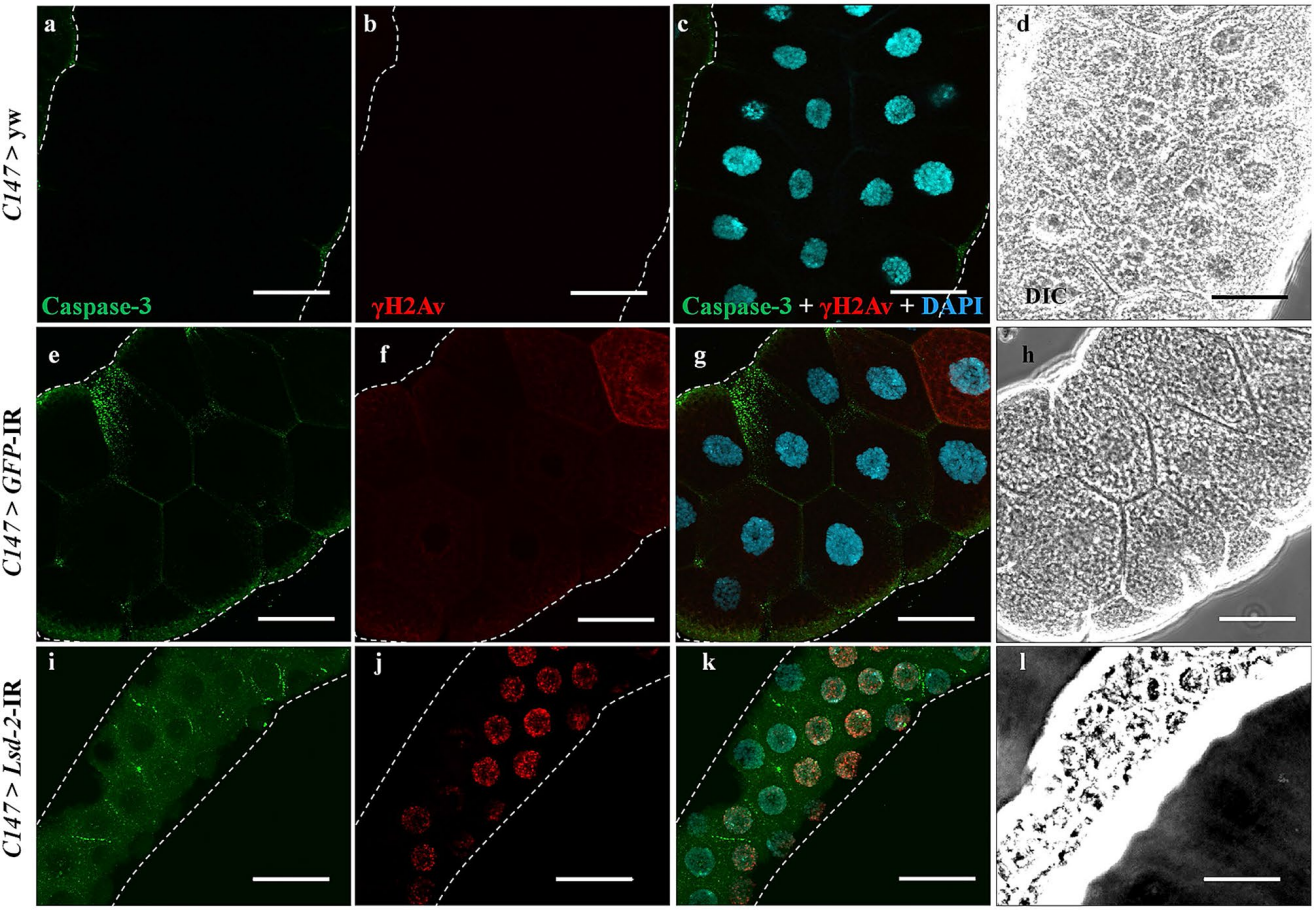
Knockdown of *Lsd-2* caused apoptosis cell death and DNA damage in salivary gland. Salivary glands from middle stage 3rd-instar larvae of controls and *Lsd-2* KD flies were stained with rabbit anti-cleaved caspase-3 antibody, followed by anti-rabbit IgG Alexa Fluor™ 488 antibody (**a**,**e**,**i**), and mouse anti-γH2Av antibody followed by anti-mouse IgG Alexa Fluor™ 594 antibody (**b**,**f**,**j**). Merged images of anti-γH2Av antibody, anti-cleaved caspase-3 antibody, and DAPI staining are shown (**c**,**g**,**k**). DIC images of salivary glands from 3rd-instar larvae (**d**,**h**,**l**) are shown. The images in the figure are representative images of 30 salivary glands. The dotted line indicates the salivary gland area. Scale bar, 50 μm. Genotypes: (**a**–**d**) +; *C147*-GAL4/+; +, (**e**–**h**) +; *C147*-GAL4/+; UAS-*GFP*-IR/+, (**i**–**l**) +; *C147*-GAL4/UAS*-Lsd-2-*IR_556–888_; +. *DIC* differential interference contrast.

To reveal that salivary gland cells induce DNA damage by the depletion of LSD-2, we examined the expression of histone variant H2Av phosphorylated at Ser137 (γH2Av), a marker for an early signal of DNA damage induced by replication stress^33,34^. As shown in Fig. 8j, salivary glands from middle stage 3rd-instar larvae of *Lsd-2* KD flies showed a markedly increased number of γH2Av-positive cells, suggesting that the knockdown of *Lsd-2* on salivary gland cells led to DNA damage. Next, we examined whether *Lsd-2* KD enhanced apoptotic cell death. After immunostaining, salivary glands from middle stage 3rd-instar larvae of *Lsd-2* KD flies showed significantly higher cleaved caspase-3 signals than the control flies (Fig. 8a,e,i).

We further stained the salivary glands with amino-actinomycin D (7-AAD), which was used to stain dead cells. We found large numbers of 7-AAD-positve cells in the salivary gland from the late stage 3rd-instar larvae of *Lsd-2* KD flies (Supplementary Fig. S6d–f). These results suggest that many salivary gland cells of *Lsd-2* KD flies died or lost normal function. Furthermore, we also stained the salivary glands of late stage 3rd-instar larvae of *Lsd-2* KD flies with a cell mask, which is a marker for the cell membrane. Compared to the control flies, the cell membranes of the salivary gland cells were distinctly disrupted in the *Lsd-2* KD flies (Supplementary Fig. S6a–c). Our data indicate that *Lsd-2* dysfunction causes abnormal cell morphology and apoptotic cell death in response to DNA damage.

### LSD-2 controls the translocation of Myc from the cytoplasm into the nucleus of salivary gland cells

**Figure 9:**
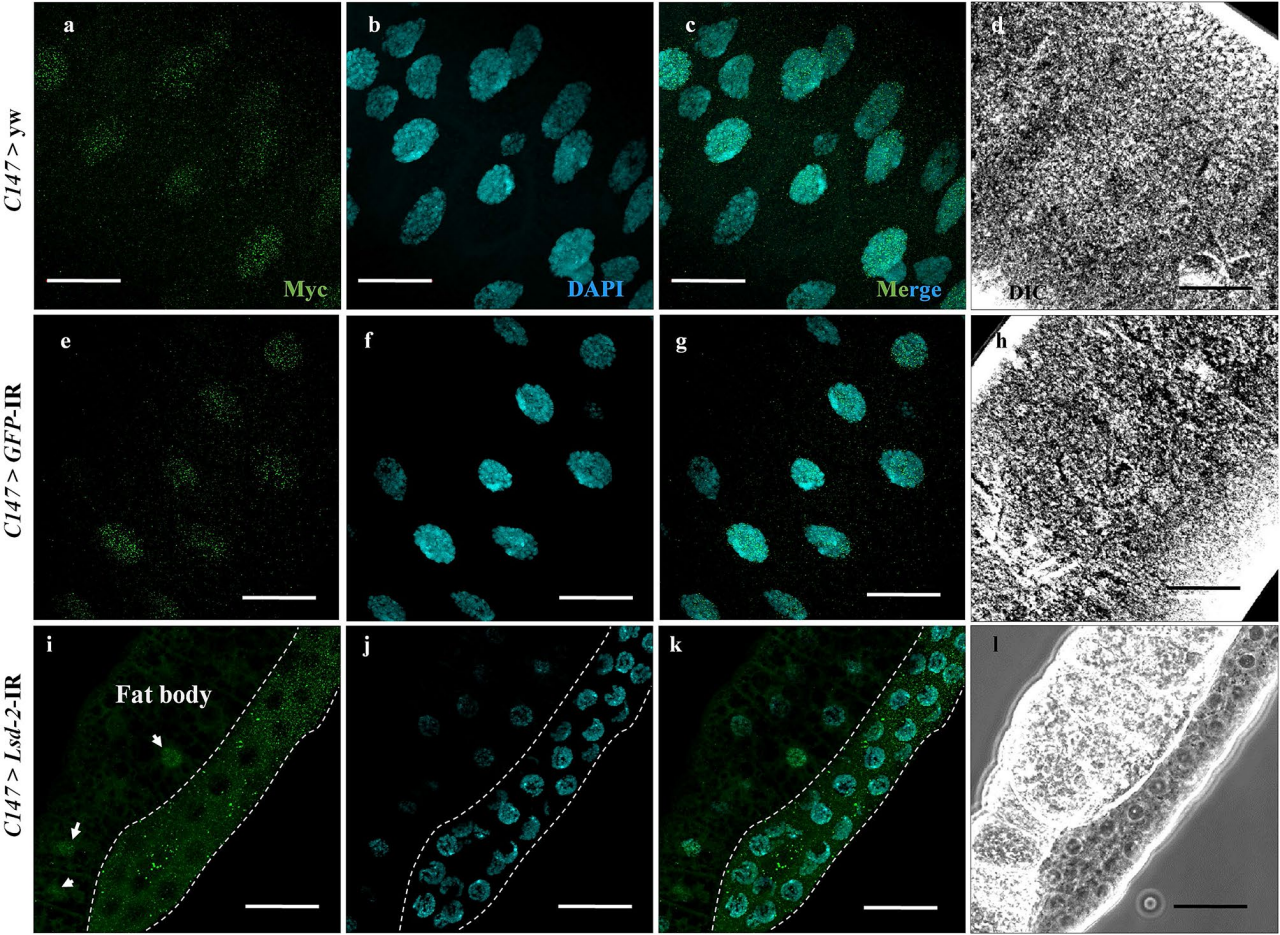
*Lsd-2* KD suppressed the translocation of dMyc from cytoplasm into the nucleus of the salivary gland cells. Salivary glands from middle stage 3rd-instar larvae of controls and *Lsd-2* KD flies were stained with mouse anti-Myc antibody, followed by anti-mouse IgG Alexa Fluor™ 488 antibody (**a**,**e**,**i**) and DAPI (**b**,**f**,**j**). Merged images of DAPI and anti-Myc antibodies are shown (**c**,**g**,**k**). DIC images of salivary glands from 3rd-instar larvae (**d**,**h**,**l**) are shown. Dotted lines surround the salivary gland area. The arrows indicate the nuclei of fat body cells used as an internal control. The images in the figure are representative images of 26 salivary glands. Scale bar, 50 μm. Genotypes: (**a**–**d**) +; *C147*-GAL4/+; +, (**e**–**h**)+; *C147*-GAL4/+; UAS-*GFP*-IR/+, (**i**–**l**) +; +; *C147*-GAL4/UAS*-Lsd-2-*IR_556–888_; +. *DIC* differential interference contrast.

In *Drosophila*, dMyc inhibits cell death triggered by JNK signaling and plays an essential role in the regulation of cell growth and cell size^35,36^. dMyc also positively controls endoreplication in *Drosophila* salivary gland cells^37,38^. Immunostaining with anti-dMyc antibody demonstrated that dMyc protein was located in the nucleus of salivary gland cells from the middle stage 3rd-instar larvae of control flies (Fig. 9a,e and Supplementary Video S1a,b). In contrast, dMyc protein was found in the cytoplasm but not in the nucleus of *Lsd-2 KD* flies (Fig. 9i and Supplementary Video S1c), whereas it was detected in the nucleus of fat body cells used as an internal control. Therefore, *Lsd-2-*KD suppressed the translocation of dMyc from the cytoplasm into the nucleus.

To confirm the genetic interaction between *Lsd-2* and *dMyc*, we generated *Lsd-2* KD flies that overexpressed dMyc or GFP as a control. Under the influence of dMyc overexpression, the atrophied salivary gland phenotypes induced by *Lsd-2* dysfunction were ameliorated compared to the control flies (Supplementary Fig. S7). These results indicate that LSD-2 expression is essential for the translocation of dMyc to the salivary glands, leading to a controlled JNK signaling pathway.

## Discussion

PLIN2 is expressed in adipose tissue and almost all other cell types, including hepatocytes and macrophages^39^. In *Drosophila*, LSD-2 functions as a conserved surface-associated module of lipid droplets in all cells and muscles and plays a pivotal role in lipolysis and lipid droplet transport^40^. In addition, LSD-2 in the wing pouch of the wing imaginal disc plays a role in neutral lipid accumulation and wing development by regulating FoxO-dependent cell death^41,42^. Previous studies have demonstrated that LSD-2 localizes in the cytoplasm of salivary gland cells; however, the function of LSD-2 in salivary gland development has hardly been reported^12,43^. In the present study, we found that LSD-2 is expressed at high levels in specific areas of the salivary gland, in the intracellular lipid droplets of secretory cells and the lumen of the tube in which duct cells reside, which connects the secretory cells to the larval mouth (Fig. 1). Several studies have implied that ectopic expression of PLIN2 promotes lipid accumulation and lipid droplet formation in non-adipocytes of mammals. Grönke et al*.* reported that LSD-2 is enriched in fat bodies of embryos, larvae, and the female germ line, where it plays a critical role in lipid metabolism^24^. These findings encouraged us to clarify the function of LSD-2 during salivary gland development. Our results demonstrated that LSD-2 is involved in salivary gland development at the 3rd-instar larval stage, which is characterized by small nuclei, reduced DNA content, and disrupted cell structure, but not at the 2nd-instar stage. Therefore, LSD-2 might be involved in salivary gland development by regulating endoreplication at the 3rd-instar stage.

The KD of *Lsd-2* in the whole salivary gland using the *C147*-GAL4 driver may influence the formation of duct cells, and affect nutritional intake. If this hypothesis is correct, these effects could lead to a reduction in TAG content, which is accumulated via larval food intake and disrupts the formation of the puparium. Ultimately, these effects might affect the development of salivary glands and the life cycle of *Drosophila*. However, the present study showed that depletion of LSD-2 expression increased TAG content slightly in the whole body and significantly in the salivary glands of the 3rd-instar larvae (Fig. 4g,h). In addition, whole body weight and lifespan were not affected by *Lsd-2* KD, specifically in the salivary gland (data not shown). Therefore, reduced LSD-2 expression did not affect the food intake of larvae or puparium formation.

The main function of LSD-2 is to protect lipid droplets from lipolysis mediated by a lipase, Brummer^44^. Grönke et al. also showed that adult *Lsd-2*-mutant flies showed significantly reduced TAG levels^24^. Furthermore, Fauny et al. demonstrated that neutral lipid accumulation in the wing disc was severely decreased in *Lsd-2* mutants, but was elevated after *Lsd-2* overexpression^41^. Bi et al*.*^12^ demonstrated that *plin2*-mutant flies have smaller lipid droplets in larval fat body cells than wild*-*type flies. Therefore, the observations in the present study, that is, increased TAG content and size/number of lipid droplets in the salivary glands of *Lsd-2* KD flies, indicate that lipolysis on the lipid droplets may be suppressed in this case (Fig. 4c,h). To clarify this, by analyzing the expression of lipases *bmm* and *hsl* using RT-qPCR, we found that the mRNA levels of these lipases were significantly decreased in the salivary glands of *Lsd-2* KD flies (Fig. 4j). Moreover, the reporter assay by monitoring the GFP signal as a downstream reporter of the *bmm* promoter also demonstrated decreased expression of *bmm* (Fig. 4k,l,o). Therefore, lipolysis in the *Lsd-2* KD salivary glands may be interrupted by the suppression of lipase expression.

A previous study implied that PLIN1 might have the same function as PLIN2 in the fat body of *plin2-*mutant flies^12^. Thus, the excessive intracellular lipid droplets in *Lsd-2* KD salivary gland cells might be due to overcompensation after LSD-1 expression. To test this hypothesis, we established *Lsd-2* KD flies on the background of *Lsd-1*^*1*^ and confirmed that the excess accumulation of intracellular lipid droplets by *Lsd-2* KD was partially decreased (Fig. 4e,h). In addition, the enhanced lipolysis in the salivary glands of *Lsd-2* KD flies overexpressing *bmm* strongly suppressed the accumulation of intracellular lipid droplets caused by *Lsd-2* depletion (Fig. 4d,h). These results reinforced our hypothesis that the increase in intracellular lipid droplets in the salivary gland cells of *Lsd-2* KD flies was due to the overcompensation of LSD-1. The increased or decreased accumulation of intracellular lipid droplets by manipulating BMM or LSD-1 expression did not affect the *Lsd-2* KD phenotype or abnormal salivary gland development (Fig. 4i). Thus, we could exclude the possibility that elevated intracellular lipid droplet accumulation in salivary gland leads to cytotoxicity, indirectly causing aberrant salivary gland development.

The organs employ various cell processes, such as proliferation and apoptosis, to fight against harmful internal and external conditions and maintain homeostasis. In several tissues, an alternative mechanism, endoreplication, is used for similar targets. The endoreplication cycle comprises only G- and S-phases, and therefore, generates polyploid cells, which are indispensable for achieving normal size and functionality of a range of tissues and organs^14^. Endoreplication also contributes significantly to the maintenance of genome integrity and tissue homeostasis. In animals, endoreplicating cells acquire resistance to DNA damage by lowering proapoptotic gene expression levels and play an essential role in regeneration^45,46^. The salivary gland of *Drosophila* is well known as a model for studying the endoreplication cycle. In the present study, LSD-2 affected the endocycle via the JNK pathway in *Drosophila*. The salivary glands of *Lsd-2* KD flies contained approximately the same number of cells as the controls (100–110 cells/lobe), despite their smaller size. However, *Lsd-2* KD did not show EdU-positive cells at 84-h AEH in the 3rd-instar larvae (Fig. 5). At 110–112-h AEH, the salivary gland cells of KD flies began to enter the endocycle, while endoreplication was completed in the salivary gland cells of the control cells. Therefore, LSD-2 depletion prevented the salivary gland cells from entering the endocycle and delayed this progression, leading to the formation of smaller salivary glands (Fig. 3). Previous studies have shown that cyclin E/CDK2 activity is the primary driver of S-phase entry, and a period of low CycE/Cdk2 activity in CycE oscillations is required for relicense-origin cells to allow successive endocycles^13,47,48^. These results indicate that abnormal endoreplication might be related to the misregulation of CycE expression in the salivary glands of *Lsd-2* KD flies. We found that CycE expression in early 3rd-instar larvae of *Lsd-2* KD flies was higher in the salivary glands. Continuous overexpression of CycE in the salivary glands delays endocycle progression, resulting in small salivary glands with nuclei that have undergone little DNA replication, or in ovarian follicle cells blocking polyploidization^17,49,50^. Therefore, low progression of the endocycle in the salivary glands of *Lsd-2* KD flies was caused by the overexpression of CycE.

The delay in cell entry into the S-phase in KD flies might be associated with DNA damage induced by replication stress^34^. Our results demonstrated that the salivary gland showed an extremely high level of γH2Av protein, a marker of DNA damage (Fig. 8). We also found that the salivary glands of *Lsd-2* KD flies displayed an increase in ROS production, followed by complete disruption of the cell structure, skeleton, and membrane (Fig. 6 and Supplementary Fig. S6). These data encouraged us to hypothesize that the cell death program was activated after LSD-2 depletion in the salivary glands. As expected, we found increased signals for anti-cleaved caspase-3 antibody and 7-AAD, confirming that the salivary gland cells in the *Lsd-2* KD flies had died (Fig. 8 and Supplementary Fig. S6). These results suggest that LSD-2 dysfunction disrupted cell morphology and apoptotic cell death in response to DNA damage in the 3rd-instar larvae.

Development of the *Drosophila* salivary gland is achieved primarily via the endoreplication cycle. Consequently, salivary gland tissue is mainly composed of polyploid cells^50^. This development is regulated by the JNK signaling pathway, which has a complicated relationship with tumorigenesis^51^. JNK activation is frequently associated with stimulation of cell death or inhibition of tumor growth. However, contradictory results, such as JNK activation leading to the promotion of cell proliferation or tumor formation, have also been reported^52^. Our data indicated that the p-JNK signal translocated into the nuclei of the 3rd-instar larval salivary glands of *Lsd-2* KD flies, suggesting the activation of the JNK pathway, that induced cell death in the salivary glands of the KD flies (Fig. 8).

A previous study illustrated that dMyc suppresses, whereas a lack of dMyc promotes the ectopic activation of JNK signaling-induced cell death^35^. In addition, dMyc regulates cell growth and cell size^36^. Furthermore, it is known to control endoreplication in *Drosophila* salivary gland cells^37,38^. Therefore, we investigated the involvement of Myc in the increase of cell death and atrophy of endoreplication of the *Drosophila* salivary gland. Our results showed that dMyc dramatically inhibited translocation from the cytoplasm into the nucleus in *Lsd-2* KD flies (Fig. 9). Therefore, LSD-2 depletion prevents dMyc activation, leading to the induction of early cell death in the salivary gland.

In conclusion, KD of *Lsd-2* in the salivary glands of *Drosophila* induced misregulation of lipid droplet accumulation by overcompensation of LSD-1. LSD-2 depletion might cause delayed and/or prohibited endoreplication by inducing the misexpression of CycE in the early stage and DNA damage. These events might lead to ROS accumulation and activation of the JNK pathway in the middle stage salivary gland of 3rd-instar larvae. Consequently, the cell structure of the late 3rd-instar larval salivary gland in *Drosophila* was completely destroyed by the cell death program*.* Our data implied that LSD-2 may regulate both endoreplication and cell death in *Drosophila* salivary glands by controlling Myc expression. These results demonstrated a novel function of LSD-2 in the regulation of development in the salivary gland and provide the first evidence regarding the function of LSD-2 in endocycle progression. Therefore, further experiments should be conducted to elucidate these mechanisms.

## Methods

### Fly stocks

Fly stocks were reared at 25 °C on standard food. The wild type strain used was yw. Transgenic flies carrying UAS-*Lsd-2*-IR_2128–2148_ and UAS-*Lsd-2-*IR_556–888_ were obtained from the Bloomington Stock Centre at Indiana University, USA, and the Vienna Drosophila Resource Centre, respectively, and possessed inverted repeats of the DNA downstream to the UAS sequence, to generate dsRNA that targets the region of *Lsd-2* from nucleotides 2128 to 2148 and 556 to 888, respectively. UAS-*Lsd-1*-OV, and *Lsd-1*^*1*^ flies were gifts from Prof. Ronal P. Kühnlein (University of Graz, Austria)^24^. RNAi line carrying UAS-*GFP*-IR for dsRNA control was obtained from Bloomington Stock Centre. GFP is a protein that isolated from the jellyfish, and much less harmful when expressed in *Drosophila* cells. All other flies were provided by the Kyoto Stock Centre at the Kyoto Institute of Technology, Japan, or by the Bloomington Stock Centre.

### Flip-out experiment

RNAi clones in the salivary gland and fat body were generated using a flip-out system^53^. *hs-flp*; *Act5*C > FRT y FRT > GAL4; UAS-GFP fly was a gift from Prof. Masamitsu Yamaguchi (Kyoto Institute of Technology, Japan). *hs-flp*; *Act5*C > FRT y FRT > GAL4; UAS-GFP female flies were crossed with UAS-*Lsd-2*-IR to generate random GFP-positive clones. Flip-out was induced by heat shock (45 min at 37 °C) 24–48 h after egg laying.

### Immunostaining

Five male and female transgenic flies were mated and kept for 1 day at 25 °C, after which they were transferred to a new tube containing standard food for 1 h to deposit eggs, to obtain a synchronized larval age. During the desired larval growth period, salivary glands were collected for assays. Approximately 20–30 salivary glands were fixed with 4% paraformaldehyde in PBS and stained as previously described. The following primary antibodies were used: rat anti-LSD-2 (at a ratio of 1:4000), kindly provided by Dr. Anne Ephrussi (European Molecular Biology Laboratory, Heidelberg, Germany), rabbit anti-cleaved caspase-3 IgG (1:800, Cell Signaling Technology), mouse anti-Myc (at a ratio of 1:400, Developmental Studies Hybridoma Bank USA), mouse anti-γH2Av (homolog of human histone variant; at a ratio of 1:800, Developmental Studies Hybridoma Bank), and guinea pig anti-cyclin E (at a ratio of 1:200, a gift from Prof. Jared Nordman, Vanderbilt University, USA). Samples were then incubated with secondary antibodies labeled with either Alexa Fluor 488 or Alexa Fluor 594 (1:800, Molecular Probes, Invitrogen, USA). EdU labeling was performed according to the manufacturer’s instructions (Molecular Probes). For nuclei staining, 4′,6-diamidino-2-phenylindole was used. Nile red was used to stain lipid droplets. The samples were mounted in Vectashield^®^ mounting medium (Vector Laboratories, Japan). The samples were inspected using a confocal laser microscope (FLUOVIEW FV10i, Olympus, Tokyo, Japan), and the fluorescence intensities were analyzed using MetaMorph software (version 7.7.7.0; Molecular Devices, USA).

### Triglyceride assays

TAG content was measured using an Infinity Triglyceride Assay Kit (Thermo Fisher Scientific, MA, USA), as described previously^34^. Protein concentration was measured using the Bradford assay (Bio-Rad Protein Assay Kit II, Bio-Rad Laboratories, Inc., CA, USA) to adjust the sample amount.

### Evaluation of phenotypes

Approximately 20–30 salivary glands were fixed with 4% paraformaldehyde in PBS. The samples were then stained with DAPI at 25 °C for 30 min. After reacting with DAPI, salivary glands were mounted on a glass slide in Vectashield mounting medium (Vector Laboratories, Tokyo, Japan), and then inspected using a fluorescence FV10i microscope (Olympus, Tokyo, Japan) at the same magnification and intensity. The cell number and perimeter of each lobe of the whole salivary glands were calculated to assess cell number and size.

For nucleic cells, the perimeter of the nucleic area of the salivary gland secretory cells was measured to assess the nuclei of salivary gland cells.

### Quantification of DNA content

A confocal laser microscope (FLUOVIEW FV10i, Olympus, Tokyo, Japan) was used to take 60× magnification images and the same intensity through nuclei in the salivary gland or fat body. The fluorescence intensity of single nuclei was calculated using MetaMorph software (version 7.7.7.0; Molecular Devices, USA). The average ratio intensity of the nucleic area of the salivary gland secretory cells to that of fat body cells was also calculated to assess the nuclei of salivary gland cells.

### Quantitative reverse transcription PCR (RT-qPCR)

Total RNA was extracted from salivary glands using standard Qiazol reagent (Qiagen, Germany), followed by purification using a Qiagen RNeasy^®^ Kit. cDNA was synthesized using a SimpliAmp Thermal Cycler (Life Technologies, Singapore). RT-qPCR was performed using FastStart Essential DNA Green Master Mix (Roche, Germany) on a LightCycler^®^ 96 (Roche). *Rp49* was used as the internal control. The sequences of gene-specific primers for *rp49* and *Lsd-2* were as follows: *rp49* forward: 5′-ACCAGCTTCAAGATGACCATCC-3′ and reverse: 5′-CTTGTTCGATCCGTAACCGATG-3′, *Lsd-2*^54^ forward: 5′-GGAACAACTGGCAATGGAAC-3′ and reverse: 5′-CGGGCAATTTGATGATCC-3′; *bmm* forward: 5′-GGCAATGGGAACAACTGAAC-3′ and reverse: 5′-TTGATCGGGCAATTTGATGATCC-3′; *hsl* forward: 5′-GCAGGAACAACTGATGGAAC-3′ and reverse: 5′-GCAACGGGCAATTTGATGATCC-3′; *cycE* forward: 5′-ATGAACTGGGGAACCATGGAAC-3′ and reverse: 5′-TGGCCGAATTTGATGATCC-3′.

### Statistical analysis

The experiments were repeated at least three times. Data are expressed as mean ± standard deviation. The statistical significance of differences was assessed using an unpaired *t-*test, two-tailed *t-*test, or one-way analysis of variance (ANOVA), as appropriate. Statistical significance was set at* P* < 0.05.

## Supplementary Information

Supplementary Figures.

Supplementary Video 1.

Supplementary Video 2.

Supplementary Video 3.
