# Supplementary material for: Dysfunction of lipid storage droplet-2 suppresses endoreplication and induces JNK pathway-mediated apoptotic cell death in Drosophila salivary glands
Source: Sci Rep. 2022 Mar 11;12:4302. doi: 10.1038/s41598-022-08299-6 (PMC8917166; doi:10.1038/s41598-022-08299-6)
Supplement: Supplementary file 1 — Supplementary Figures. [file 41598_2022_8299_MOESM1_ESM.pdf]

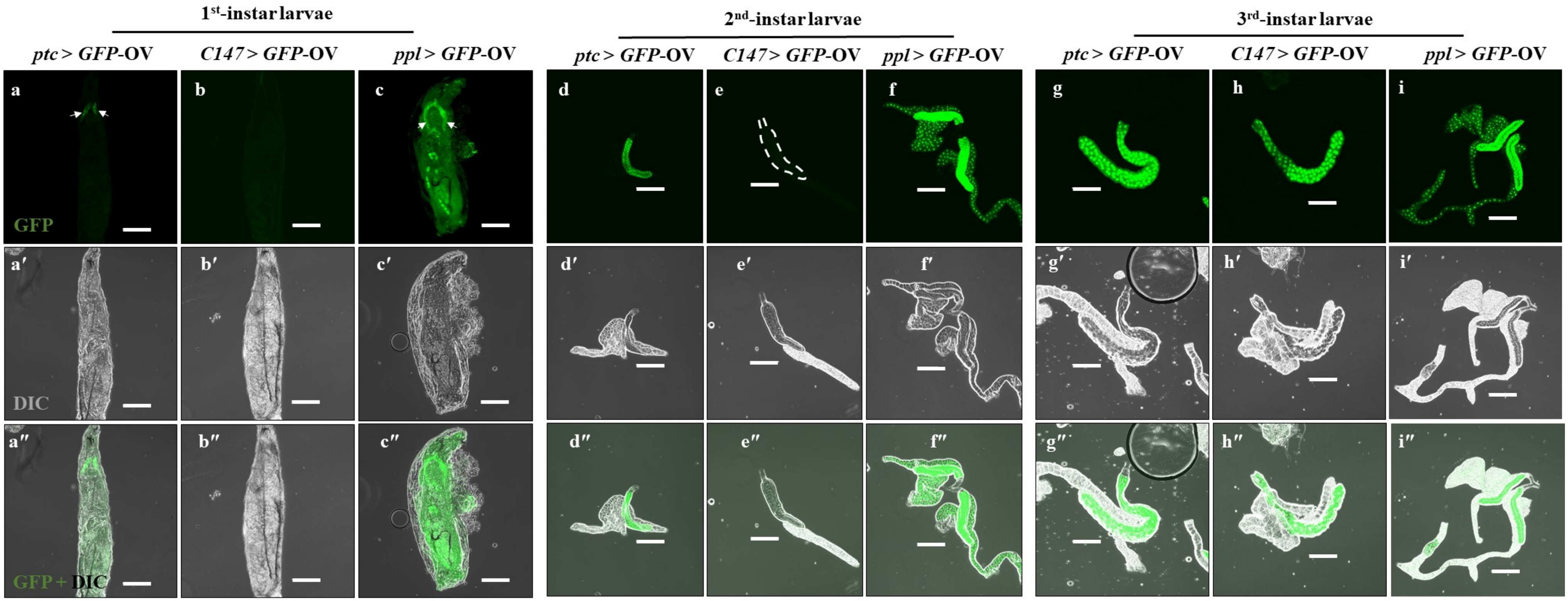

Figure S1: Regions in which GAL4 was expressed in the *ptc*-GAL4, *C147*-GAL4 and *ppl*-GAL4 driver line in the various stages of *Drosophila* larvae. The *ptc*-GAL4 and *ppl*-GAL4 driver, that expressed GAL4 in salivary gland, and fat body and salivary gland of *Drosophila* larvae, respectively, while *C147*-GAL4 driver induced GAL4 in 3rd-instar larvae of salivary gland. To confirm the regions in which GAL4 protein driven by the *ptc, ppl* and *C147* promoter were expressed, *ptc*-GAL4 *ppl*-GAL4 and *C147*-GAL4 driver lines were crossed with flies possessing the *UAS-GFP* gene. The expression of GFP in larvae was visualized by fluorescence imaging (**a-i**). DIC images of 1st-instar and salivary glands larvae (**a′-i′**) are shown. Merged image of GFP and DIC (**a″-i″**) are shown. The arrowhead and dotted line indicate the salivary gland area of larvae. Scale bar, 200 μm. Genotype: *ptc*-GAL4/+; +; UAS-*GFP*/+ (**a–a″, d–d″, g–g″**), *C147*-GAL4/+; +; UAS-*GFP*/+ (**b–b″, e–e″, h–h″**), *ppl*-GAL4/+; +; UAS-*GFP*/+ (**c–c″, e–e″, i–i″**). DIC, differential interference contrast.

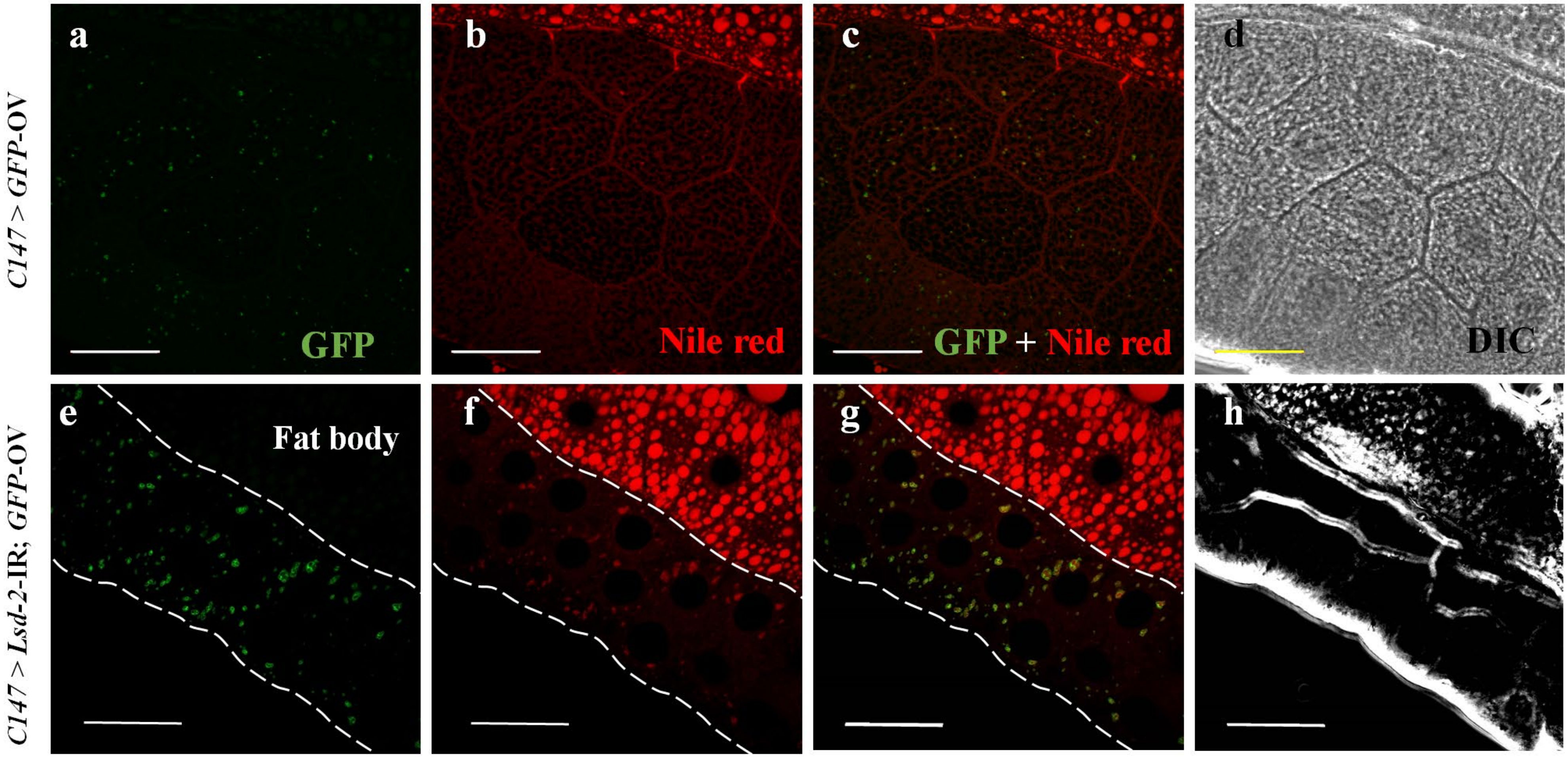

Figure S2. Regions in which GAL4 was expressed in the *C147*-GAL4 driver line in 3rd-instar larvae. To confirm the regions in which GAL4 protein driven by the *C147* promoter was expressed, the *C147*-GAL4 driver line was crossed with *Lsd*-2-IR; *GFP*-OV and GFP overexpression (*GFP*-OV) flies were used as the control. The 3rd-instar salivary glands and fat body were stained with Nile red to detect lipid droplets. GFP expression and Nile red staining in the salivary gland and fat body of 3rd-instar larvae were visualized by fluorescence imaging (**a-b** and **e-f**). Merged image of GFP and Nile red results (**c-g**). Dotted lines indicate the salivary gland area. DIC images of salivary glands from 3rd-instar larvae (**d-h**) are shown. Scale bar, 50 μm. Genotype: (**a-d**) +; *C147*-GAL4/+; UAS-*GFP*/+, (**e-h**) +; *C147*-GAL4/UAS-*Lsd-2*-IR; UAS-*GFP*/+. DIC, differential interference contrast.

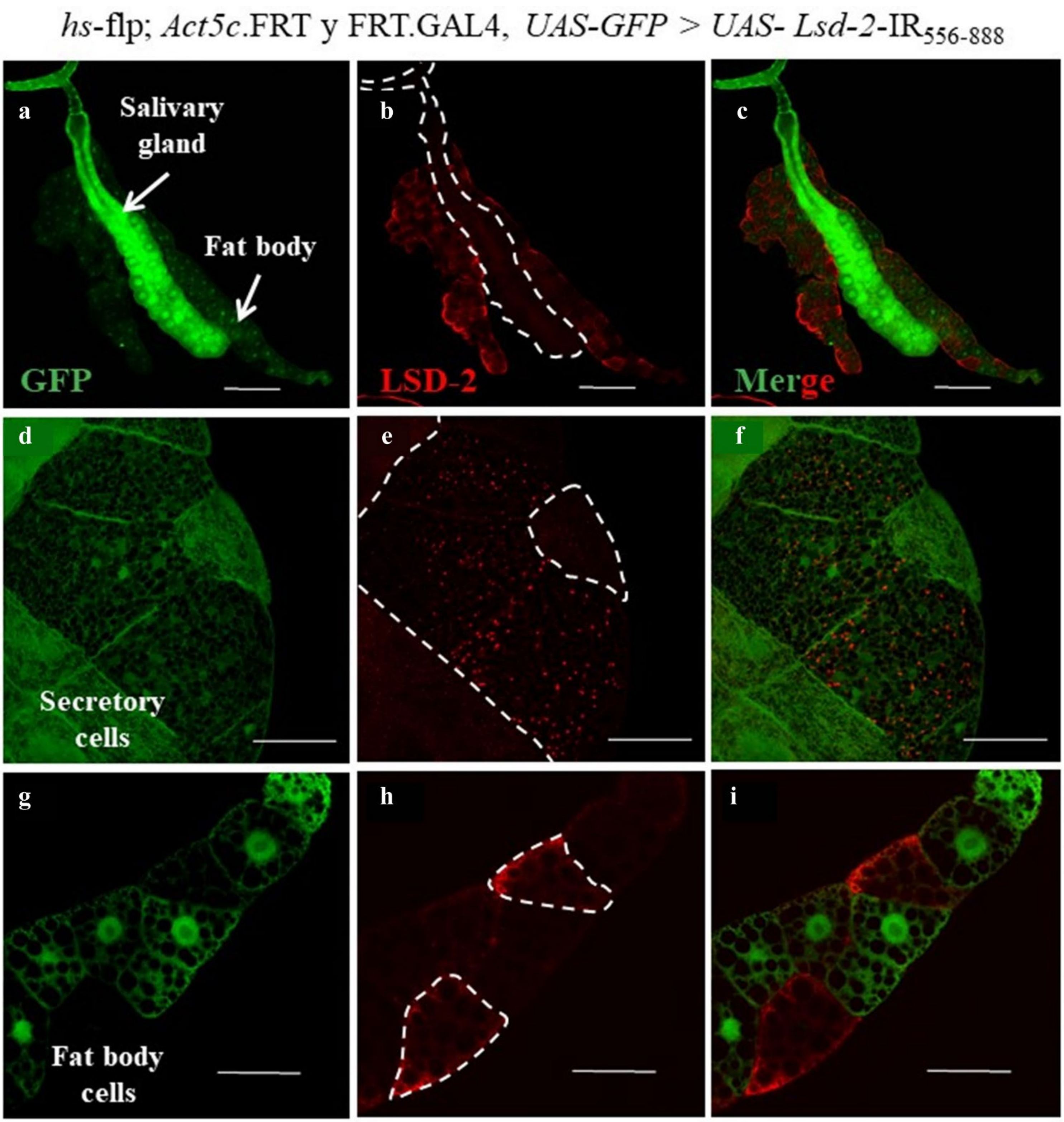

Figure S3. Expression of *Lsd-2* dsRNA reduced LSD-2 levels in the salivary gland and fat body.

In the flip-out experiment using *Act5c* driver, salivary gland and fat body were stained with anti-LSD-2 antibody, followed by anti-rabbit IgG Alexa Fluor^{TM} 594 antibody (**b, e,** and **h**). Cells expressing *Lsd-2* dsRNA were marked with GFP (**a, d**, and **g**). The anti-LSD-2 antibody and GFP signals were merged (**c, f,** and **i**). Dotted line indicates the GFP-positive region in which *Lsd-2* dsRNA is expressed. Scale bar, 200 μm (**a–c**); 50 μm (**d–i**).

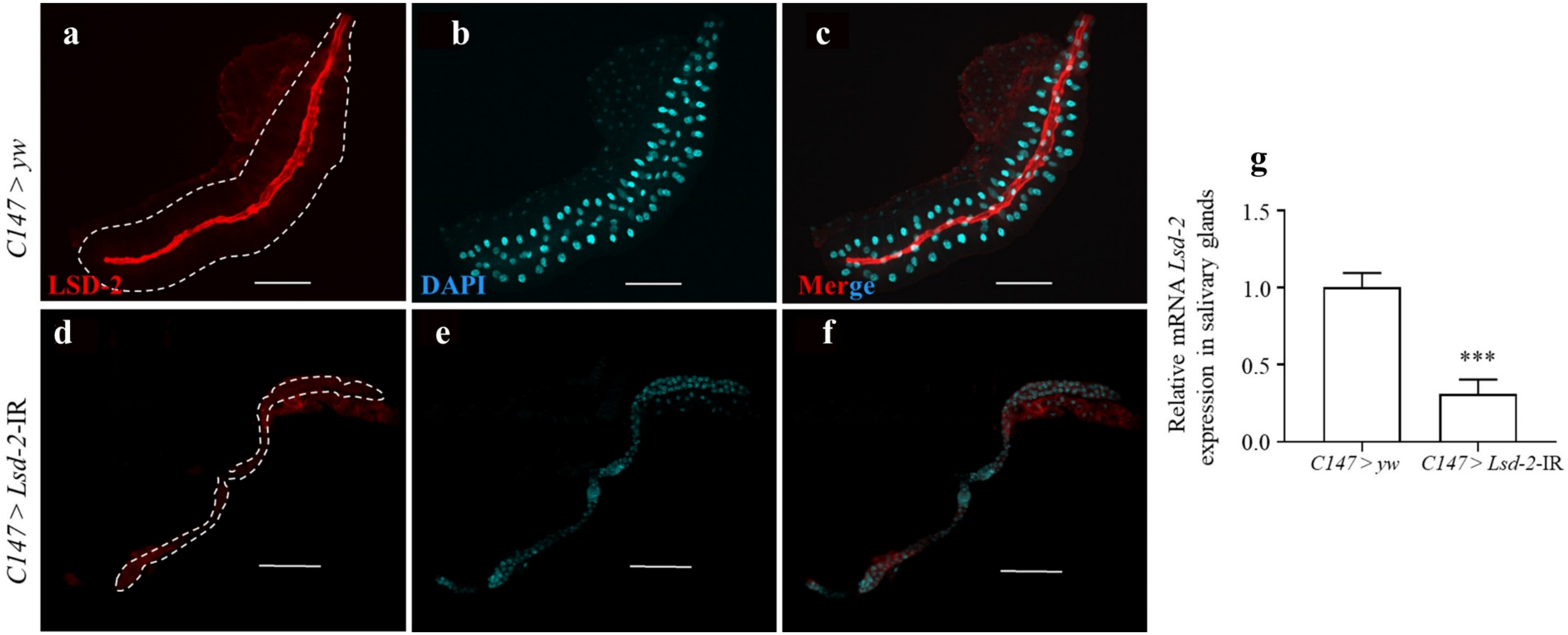

Figure S4. Induction of *Lsd-2* RNAi led to reduced expression of LSD-2. Salivary glands of control and *Lsd-2* KD flies were stained with anti-rat-LSD-2 antibody, followed by anti-rat IgG Alexa Fluor™ 594 antibody (**a** and **b**), and DAPI to visualize DNA (**b** and **e**). Both images of DAPI staining and immunostaining were merged (**c** and **f**). The images in the figure are representative of images from 30 salivary glands. *Lsd-2* mRNA levels in the salivary glands of 3rd-instar larvae of control and *Lsd-2* KD flies were analyzed using RT-qPCR (**g**, n=4). Dotted line indicates the salivary gland area. Scale bar, 200 µm. Genotypes: (**a–c**) +; *C147*-GAL4/+; +, (**d–f**) +; *C147*-GAL4/UAS-*Lsd-2*-IR$_{556\text{-}888}$/; +.

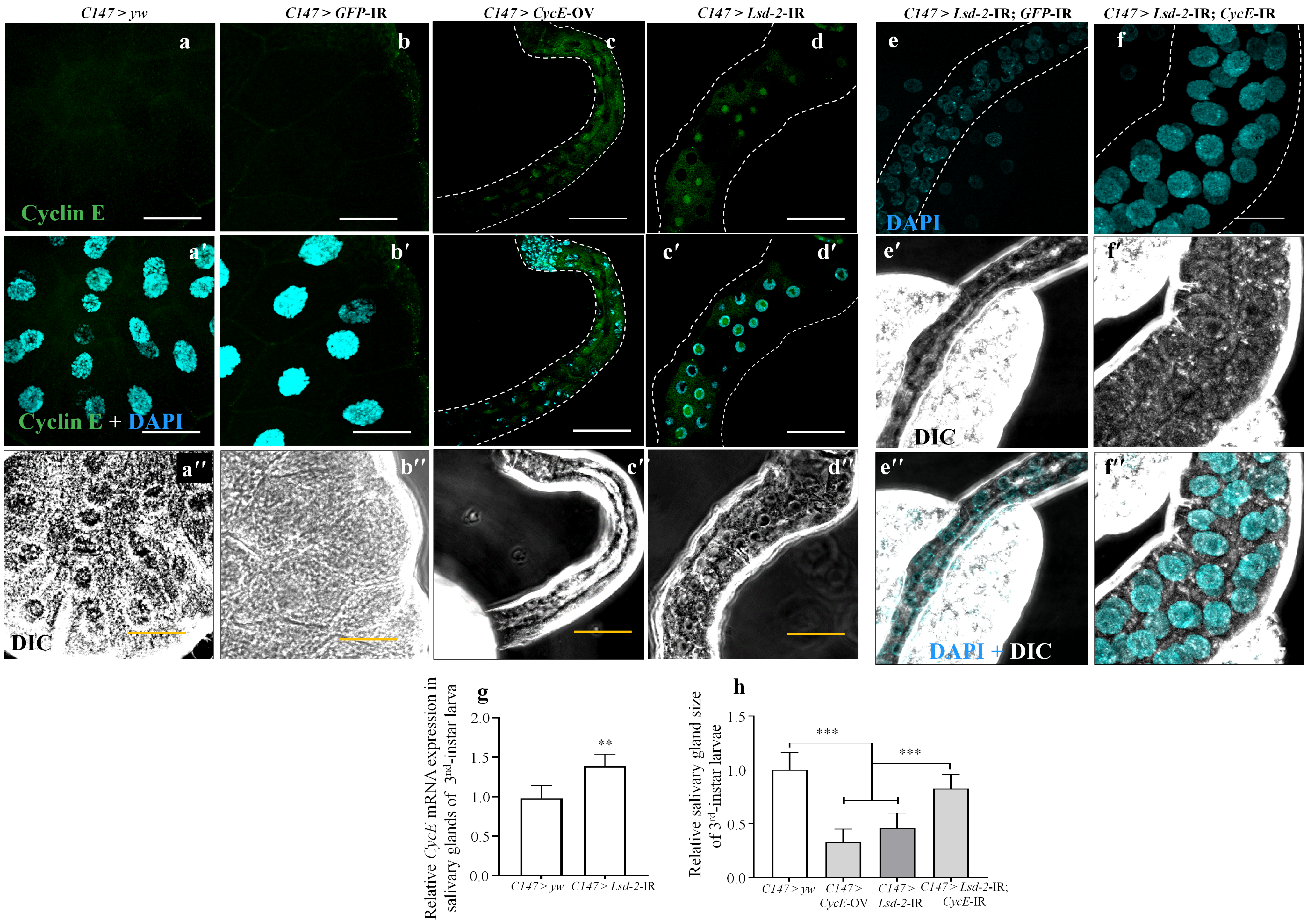

C147 > yw
C147 > GFP-IR
C147 > CycE-OV
C147 > Lsd-2-IR
C147 > Lsd-2-IR; GFP-IR
C147 > Lsd-2-IR; CycE-IR
a
b
c
d
e
f
Cyclin E
DAPI
a′
b′
c′
d′
e′
f′
Cyclin E + DAPI
DIC
a′′
b′′
c′′
d′′
e′′
f′′
DIC
DAPI + DIC
g
Relative CycE mRNA expression in salivary glands of 3rd-instar larva
2.0
1.5
1.0
0.5
0.0
**
C147 > yw
C147 > Lsd-2-IR
h
Relative salivary gland size of 3rd-instar larvae
1.5
1.0
0.5
0.0
***
***
C147 > yw
C147 > CycE-OV
C147 > Lsd-2-IR
C147 > Lsd-2-IR; CycE-IR

Figure S5. Knockdown of *Lsd-2* increased CycE expression in salivary glands. The early stage 3rd-instar larval salivary glands of three strains–controls (*C147* > yw and *C147* > *GFP*-IR), CycE overexpression (*C147* > *CycE*-OV), and *Lsd-2* KD (*C147* > *Lsd-2*-IR)–were stained with anti-CycE antibody, followed by anti-guinea pig IgG Alexa Fluor™ 488 antibody (**a – d)**. Merged images of anti-CycE antibody and DAPI staining are shown (**a′–d′**). DIC images of salivary glands from 3rd-instar larvae are shown (**a″–d″**). *CycE* mRNA levels in the salivary glands of early stage 3rd-instar larvae of control and *Lsd-2* KD flies were analyzed using RT-qPCR (**g**, *n*=4). The relative sizes of salivary glands of early 3rd-instar larvae of *CycE*-OV, *Lsd-2* KD and double KD of *Lsd-2* and *CycE* (**e–e″**, *C147* > *Lsd-2*-IR; *CycE*-IR) and *GFP*-IR (**f–f″**, *C147* > *Lsd-2*-IR; *GFP*-IR) flies, as compared to control flies (*C147*>yw, *C147*>*GFP*-IR) are shown (**a–a″ and f–f″**, *n*=20). Dotted line indicates the salivary gland area. Statistical significance of differences in parameters between controls and other flies was evaluated using one-way ANOVA and Student's *t*-test. **, p<0.01 ***, p<0.001. Scale bar, 50 µm. Genotypes: (**a–a″**) +; *C147*-GAL4/+; +, (**b–b″**) +; *C147*-GAL4/+; UAS-*GFP*-IR/+, (**c–c″**) +; *C147*-GAL4/UAS-*CycE*-OV; +, (**d–d″**) +; *C147*-GAL4/UAS-*Lsd-2*-$IR_{556\text{-}888}$; +, (**e–e″**) +; *C147*-GAL4/UAS-*Lsd-2*-$IR_{556\text{-}888}$; UAS-*GFP*-IR/+, (**f–f″**) +; *C147*-GAL4/UAS-*Lsd-2*-$IR_{556\text{-}888}$; UAS-*CycE*-IR/+.

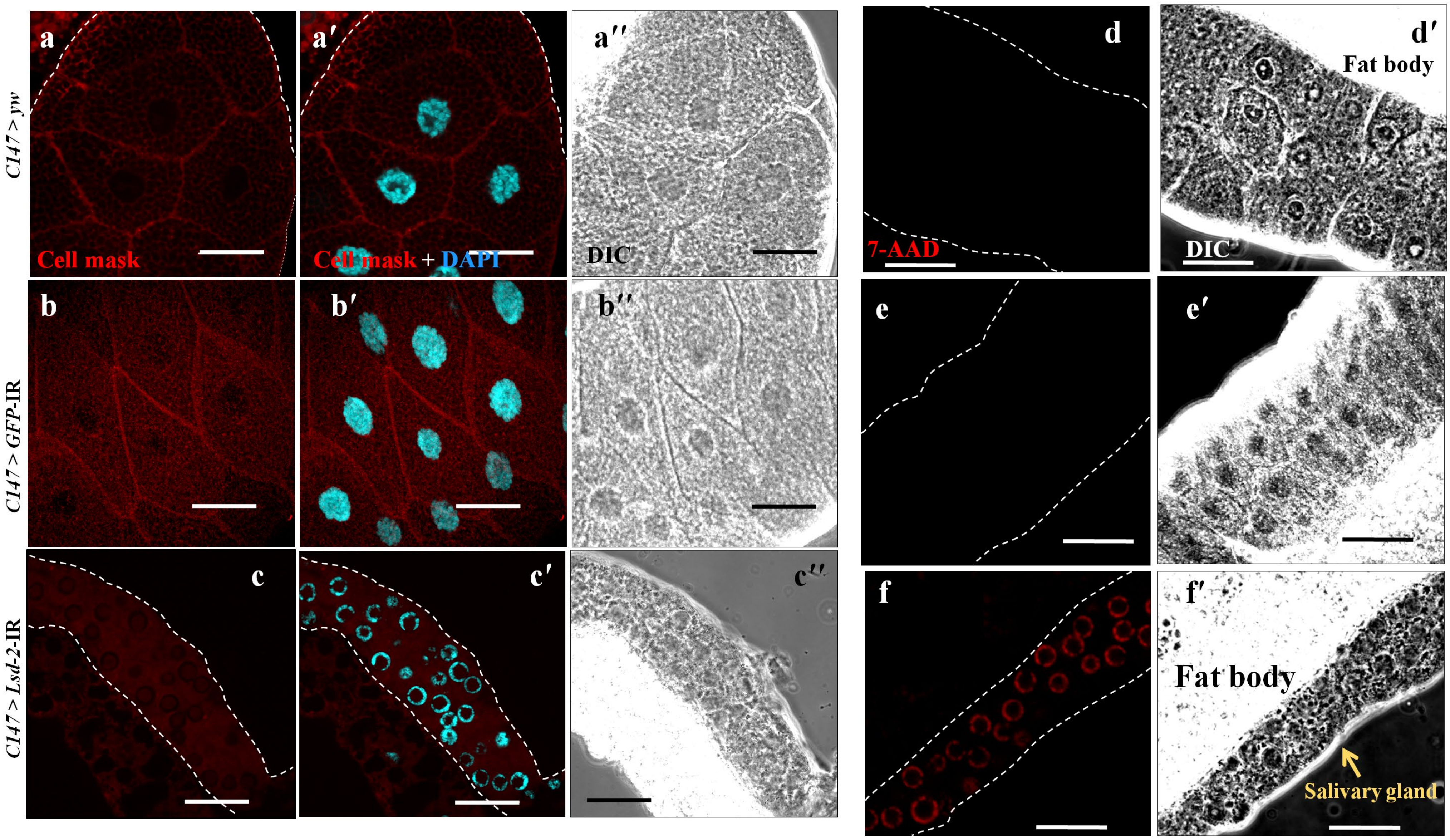

Figure S6. Knockdown of *Lsd-2* induced disruption of cell membrane and cell death in salivary gland. Salivary glands from late 3rd-instar larvae of controls and *Lsd-2* KD flies were stained with cell mask to detect the cell membrane (**a**, **b** and **c**) and with 7-AAD to visualize the DNA of dead cells (**d**, **e** and **f**). Merged images of DAPI and cell mask staining results are shown (**a′**, **b′** and **c′**). DIC images are also shown (**a″– c″** and **d′**, **f′**). The images in the figure are representative images for 26 salivary glands. Dotted lines indicate the salivary gland area. Scale bar, 50 µm. Genotypes: (**a, a′, a″** and **d, d′**) +; *C147*-GAL4/+; +, (**b, b′, b″** and **e, e′**) +; *C147*-GAL4/+; UAS-*GFP*-IR/+, (**c, c′, c″** and **f, f′**) +; *C147*-GAL4/UAS-*Lsd-2*-IR$_{556\text{-}888}$/; +.

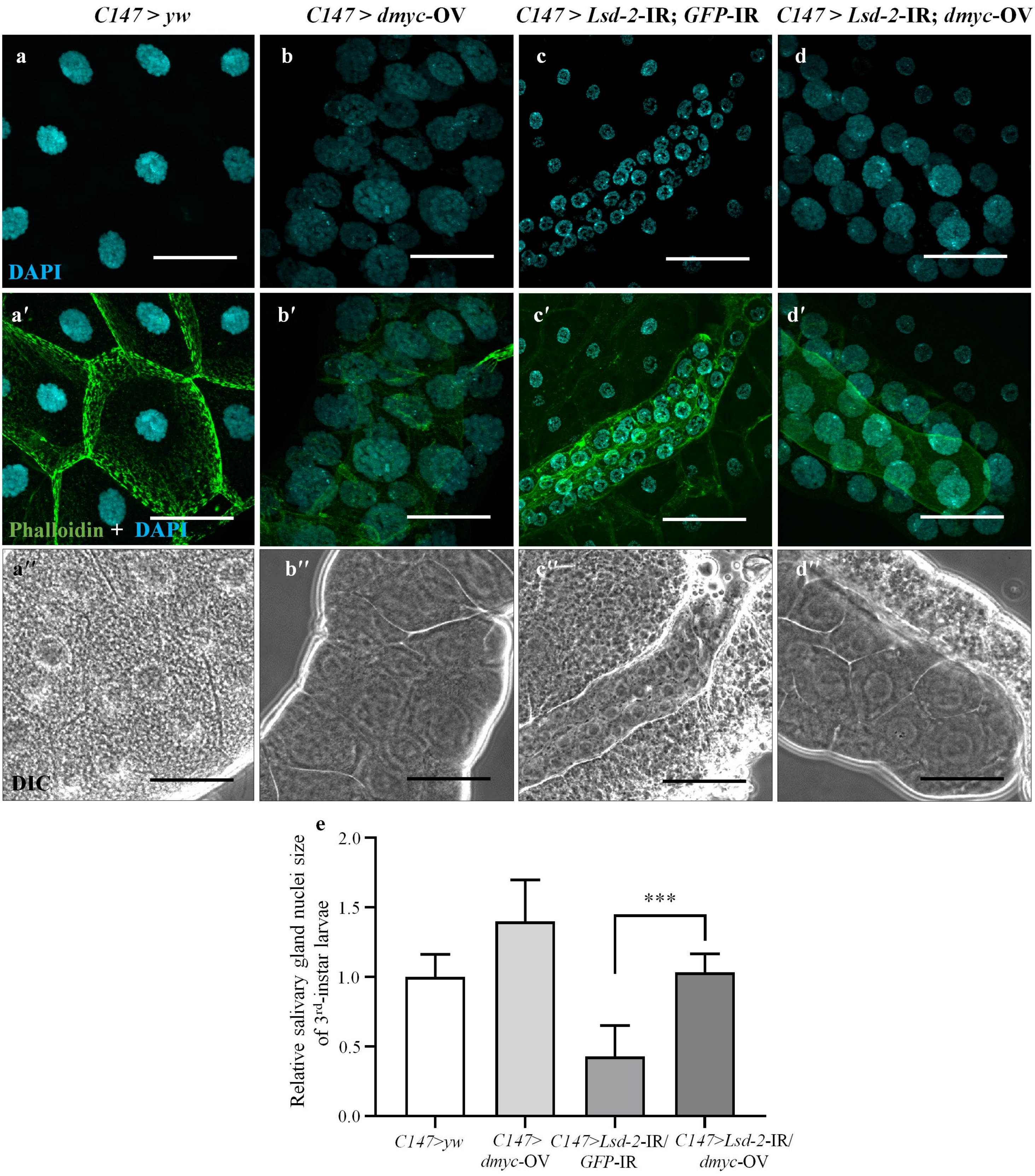

C147 > yw
C147 > dmyc-OV
C147 > Lsd-2-IR; GFP-IR
C147 > Lsd-2-IR; dmyc-OV
a
b
c
d
DAPI
a′
b′
c′
d′
Phalloidin + DAPI
a″
b″
c″
d″
DIC
e
Relative salivary gland nuclei size of 3rd-instar larvae
2.0
1.5
1.0
0.5
0.0
***
C147>yw
C147> dmyc-OV
C147>Lsd-2-IR/ GFP-IR
C147>Lsd-2-IR/ dmyc-OV

Figure S7. The *Lsd-2* knockdown phenotypes of cell skeleton disruption and DNA content reduction were rescued by overexpression of dMYC in salivary gland. The salivary glands of middle stage 3rd-instar larvae were stained with DAPI (**a–d**) to visualize the DNA. Merged images of DAPI and phalloidin, which are used to label the cell skeleton, are shown (**a′–d′**). The *dmyc*-OV possessed larger nucleus of salivary glands (**b, b′**), as compared to those in the control flies (**a, a′**; *C147* > yw). The *Lsd-2* KD phenotypes were rescued by overexpression of dMYC (**d, d′**; *C147>Lsd-2*-IR/*dmyc*-OV) but not by expression of GFP dsRNAi (**c, c′**). DIC images are also shown (**a″– d″**). The sizes of nuclei in the salivary gland and fat body were measured using ImageJ software, and the relative nucleic size of the salivary gland cells is shown (**e**, n=16). The images in the figure are representative images for 16 salivary glands. Statistical significance of differences in nuclei sizes between *Lsd*-2 KD and rescue flies was evaluated using Student's *t*-test. ***, p<0.001 Scale bar, 50 µm. Genotypes: (**a-a″**) +; *C147*-GAL4/+; +, (**b-b″**) +; *C147*-GAL4/+; UAS-*dmyc*-OV/+, (**c-c″**) +; *C147*-GAL4/UAS-*Lsd-2*-IR$_{556-888}$/+; UAS-*GFP*-IR/+, (**d-d″**) +; *C147*-GAL4/UAS-*Lsd-2*-IR$_{556-888}$/+; UAS-*dmyc*-OV/+.

Video S1 legend. ***Lsd-2*** **KD suppressed the translocation of dMyc from cytoplasm into the nucleus of the salivary gland cells.** Salivary glands from middle stage 3rd-instar larvae of controls and *Lsd-2* KD flies were stained with mouse anti-Myc antibody, followed by anti-mouse IgG Alexa Fluor™ 488 antibody (**a**–**c**). Scale bar, 50 µm. Genotypes: (a) +; *C147*-GAL4/+; +, (b) +; *C147*-GAL4/+; UAS-*GFP*-IR/+, (c) +; *C147*-GAL4/UAS-*Lsd-2*-IR$_{556\text{-}888}$; +.
